# A multimodal cell census and atlas of the mammalian primary motor cortex

**DOI:** 10.1038/s41586-021-03950-0

**Published:** 2021-10-06

**Authors:** BRAIN Initiative Cell Census Network (BICCN), Edward M. Callaway, Hong-Wei Dong, Joseph R. Ecker, Michael J. Hawrylycz, Z. Josh Huang, Ed S. Lein, John Ngai, Pavel Osten, Bing Ren, Andreas Savas Tolias, Owen White, Hongkui Zeng, Xiaowei Zhuang, Giorgio A. Ascoli, M. Margarita Behrens, Jerold Chun, Guoping Feng, James C. Gee, Satrajit S. Ghosh, Yaroslav O. Halchenko, Ronna Hertzano, Byung Kook Lim, Maryann E. Martone, Lydia Ng, Lior Pachter, Alexander J. Ropelewski, Timothy L. Tickle, X. William Yang, Kun Zhang, Trygve E. Bakken, Philipp Berens, Tanya L. Daigle, Julie A. Harris, Nikolas L. Jorstad, Brian E. Kalmbach, Dmitry Kobak, Yang Eric Li, Hanqing Liu, Katherine S. Matho, Eran A. Mukamel, Maitham Naeemi, Federico Scala, Pengcheng Tan, Jonathan T. Ting, Fangming Xie, Meng Zhang, Zhuzhu Zhang, Jingtian Zhou, Brian Zingg, Ethan Armand, Zizhen Yao, Darren Bertagnolli, Tamara Casper, Kirsten Crichton, Nick Dee, Dinh Diep, Song-Lin Ding, Weixiu Dong, Elizabeth L. Dougherty, Olivia Fong, Melissa Goldman, Jeff Goldy, Rebecca D. Hodge, Lijuan Hu, C. Dirk Keene, Fenna M. Krienen, Matthew Kroll, Blue B. Lake, Kanan Lathia, Sten Linnarsson, Christine S. Liu, Evan Z. Macosko, Steven A. McCarroll, Delissa McMillen, Naeem M. Nadaf, Thuc Nghi Nguyen, Carter R. Palmer, Thanh Pham, Nongluk Plongthongkum, Nora M. Reed, Aviv Regev, Christine Rimorin, William J. Romanow, Steven Savoia, Kimberly Siletti, Kimberly Smith, Josef Sulc, Bosiljka Tasic, Michael Tieu, Amy Torkelson, Herman Tung, Cindy T. J. van Velthoven, Charles R. Vanderburg, Anna Marie Yanny, Rongxin Fang, Xiaomeng Hou, Jacinta D. Lucero, Julia K. Osteen, Antonio Pinto-Duarte, Olivier Poirion, Sebastian Preissl, Xinxin Wang, Andrew I. Aldridge, Anna Bartlett, Lara Boggeman, Carolyn O’Connor, Rosa G. Castanon, Huaming Chen, Conor Fitzpatrick, Chongyuan Luo, Joseph R. Nery, Michael Nunn, Angeline C. Rivkin, Wei Tian, Bertha Dominguez, Tony Ito-Cole, Matthew Jacobs, Xin Jin, Cheng-Ta Lee, Kuo-Fen Lee, Paula Assakura Miyazaki, Yan Pang, Mohammad Rashid, Jared B. Smith, Minh Vu, Elora Williams, Tommaso Biancalani, A. Sina Booeshaghi, Megan Crow, Sandrine Dudoit, Stephan Fischer, Jesse Gillis, Qiwen Hu, Peter V. Kharchenko, Sheng-Yong Niu, Vasilis Ntranos, Elizabeth Purdom, Davide Risso, Hector Roux de Bézieux, Saroja Somasundaram, Kelly Street, Valentine Svensson, Eeshit Dhaval Vaishnav, Koen Van den Berge, Joshua D. Welch, Xu An, Helen S. Bateup, Ian Bowman, Rebecca K. Chance, Nicholas N. Foster, William Galbavy, Hui Gong, Lin Gou, Joshua T. Hatfield, Houri Hintiryan, Karla E. Hirokawa, Gukhan Kim, Daniel J. Kramer, Anan Li, Xiangning Li, Qingming Luo, Rodrigo Muñoz-Castañeda, David A. Stafford, Zhao Feng, Xueyan Jia, Shengdian Jiang, Tao Jiang, Xiuli Kuang, Rachael Larsen, Phil Lesnar, Yaoyao Li, Yuanyuan Li, Lijuan Liu, Hanchuan Peng, Lei Qu, Miao Ren, Zongcai Ruan, Elise Shen, Yuanyuan Song, Wayne Wakeman, Peng Wang, Yimin Wang, Yun Wang, Lulu Yin, Jing Yuan, Sujun Zhao, Xuan Zhao, Arun Narasimhan, Ramesh Palaniswamy, Samik Banerjee, Liya Ding, Dhananjay Huilgol, Bingxing Huo, Hsien-Chi Kuo, Sophie Laturnus, Xu Li, Partha P. Mitra, Judith Mizrachi, Quanxin Wang, Peng Xie, Feng Xiong, Yang Yu, Stephen W. Eichhorn, Jim Berg, Matteo Bernabucci, Yves Bernaerts, Cathryn René Cadwell, Jesus Ramon Castro, Rachel Dalley, Leonard Hartmanis, Gregory D. Horwitz, Xiaolong Jiang, Andrew L. Ko, Elanine Miranda, Shalaka Mulherkar, Philip R. Nicovich, Scott F. Owen, Rickard Sandberg, Staci A. Sorensen, Zheng Huan Tan, Shona Allen, Dirk Hockemeyer, Angus Y. Lee, Matthew B. Veldman, Ricky S. Adkins, Seth A. Ament, Héctor Corrada Bravo, Robert Carter, Apaala Chatterjee, Carlo Colantuoni, Jonathan Crabtree, Heather Creasy, Victor Felix, Michelle Giglio, Brian R. Herb, Jayaram Kancherla, Anup Mahurkar, Carrie McCracken, Lance Nickel, Dustin Olley, Joshua Orvis, Michael Schor, Greg Hood, Benjamin Dichter, Michael Grauer, Brian Helba, Anita Bandrowski, Nikolaos Barkas, Benjamin Carlin, Florence D. D’Orazi, Kylee Degatano, Thomas H. Gillespie, Farzaneh Khajouei, Kishori Konwar, Carol Thompson, Kathleen Kelly, Stephanie Mok, Susan Sunkin

**Affiliations:** 1 Systems Neurobiology Laboratories, The Salk Institute for Biological Studies, La Jolla, CA USA; 2 UCLA Brain Research and Artificial Intelligence Nexus, Department of Neurobiology, David Geffen School of Medicine, University of California Los Angeles, Los Angeles, CA USA; 3 Genomic Analysis Laboratory, The Salk Institute for Biological Studies, La Jolla, CA USA; 4 Howard Hughes Medical Institute, The Salk Institute for Biological Studies, La Jolla, CA USA; 5 Allen Institute for Brain Science, Seattle, WA USA; 6 Cold Spring Harbor Laboratory, Cold Spring Harbor, NY USA; 7 Department of Neurobiology, Duke University School of Medicine, Durham, NC USA; 8 Department of Molecular and Cell Biology, University of California Berkeley, Berkeley, CA USA; 9 Helen Wills Neuroscience Institute, University of California Berkeley, Berkeley, CA USA; 10 Center for Epigenomics, Department of Cellular and Molecular Medicine, University of California San Diego School of Medicine, La Jolla, CA USA; 11 Ludwig Institute for Cancer Research, La Jolla, CA USA; 12 Department of Neuroscience, Baylor College of Medicine, Houston, TX USA; 13 Center for Neuroscience and Artificial Intelligence, Baylor College of Medicine, Houston, TX USA; 14 Institute for Genome Sciences, University of Maryland School of Medicine, Baltimore, MD USA; 15 Howard Hughes Medical Institute, Department of Chemistry and Chemical Biology, Department of Physics, Harvard University, Cambridge, MA USA; 16 Center for Neural Informatics, Krasnow Institute for Advanced Study, George Mason University, Fairfax, VA USA; 17 Bioengineering Department, George Mason University, Fairfax, VA USA; 18 Computational Neurobiology Laboratory, The Salk Institute for Biological Studies, La Jolla, CA USA; 19 Sanford Burnham Prebys Medical Discovery Institute, La Jolla, CA USA; 20 McGovern Institute for Brain Research, Massachusetts Institute of Technology, Cambridge, MA USA; 21 Department of Brain and Cognitive Sciences, Massachusetts Institute of Technology, Cambridge, MA USA; 22 Stanley Center for Psychiatric Research, Broad Institute of MIT and Harvard, Cambridge, MA USA; 23 Department of Radiology, University of Pennsylvania, Philadelphia, PA USA; 24 Massachusetts Institute of Technology, Cambridge, MA USA; 25 Dartmouth College, Hannover, NH USA; 26 Department of Otorhinolaryngology, Anatomy and Neurobiology, University of Maryland School of Medicine, Baltimore, MD USA; 27 Division of Biological Science, Neurobiology Section, University of California San Diego, La Jolla, CA USA; 28 Department of Neurosciences, University of California San Diego, La Jolla, CA USA; 29 SciCrunch, Inc., San Diego, CA USA; 30 California Institute of Technology, Pasadena, CA USA; 31 Pittsburgh Supercomputing Center, Carnegie Mellon University, Pittsburgh, PA USA; 32 Klarman Cell Observatory and Data Sciences Platform, Broad Institute of MIT and Harvard, Cambridge, MA USA; 33 Center for Neurobehavioral Genetics, Jane and Terry Semel Institute for Neuroscience and Human Behaviors, University of California Los Angeles, Los Angeles, CA USA; 34 Department of Psychiatry and Biobehavioral Science, David Geffen School of Medicine, University of California Los Angeles, Los Angeles, CA USA; 35 Department of Bioengineering, University of California San Diego, La Jolla, CA USA; 36 Institute for Ophthalmic Research, University of Tübingen, Tübingen, Germany; 37 Center for Integrative Neuroscience, University of Tübingen, Tübingen, Germany; 38 Institute for Bioinformatics and Medical Informatics, University of Tübingen, Tübingen, Germany; 39 Bernstein Center for Computational Neuroscience, University of Tübingen, Tübingen, Germany; 40 Department of Physiology and Biophysics, University of Washington, Seattle, WA USA; 41 Division of Biological Sciences, University of California San Diego, La Jolla, CA USA; 42 Department of Cognitive Science, University of California San Diego, La Jolla, CA USA; 43 School of Pharmaceutical Sciences, Tsinghua University, Beijing, China; 44 Department of Physics, University of California San Diego, La Jolla, CA USA; 45 Bioinformatics and Systems Biology Graduate Program, University of California San Diego, La Jolla, CA USA; 46 Broad Institute of MIT and Harvard, Cambridge, MA USA; 47 Department of Genetics, Harvard Medical School, Boston, MA USA; 48 Division of Molecular Neurobiology, Department of Medical Biochemistry and Biophysics, Karolinska Institute, Stockholm, Sweden; 49 Department of Laboratory Medicine and Pathology, University of Washington, Seattle, WA USA; 50 Biomedical Sciences Program, School of Medicine, University of California San Diego, La Jolla, CA USA; 51 Howard Hughes Medical Institute, Department of Biology, MIT, Cambridge, MA USA; 52 Flow Cytometry Core Facility, The Salk Institute for Biological Studies, La Jolla, CA USA; 53 Peptide Biology Laboratories, The Salk Institute for Biological Studies, La Jolla, CA USA; 54 Molecular Neurobiology Laboratory, The Salk Institute for Biological Studies, La Jolla, CA USA; 55 Stanley Institute for Cognitive Genomics, Cold Spring Harbor Laboratory, Cold Spring Harbor, NY USA; 56 Department of Statistics and Division of Biostatistics, University of California Berkeley, Berkeley, CA USA; 57 Department of Biomedical Informatics, Harvard Medical School, Boston, MA USA; 58 University of California San Francisco, San Francisco, CA USA; 59 Department of Statistics, University of California Berkeley, Berkeley, CA USA; 60 Department of Statistical Sciences, University of Padova, Padua, Italy; 61 Division of Biostatistics, School of Public Health, University of California Berkeley, Berkeley, CA USA; 62 Department of Data Sciences, Dana-Farber Cancer Institute, Boston, MA USA; 63 Department of Applied Mathematics, Computer Science and Statistics, Ghent University, Ghent, Belgium; 64 Department of Computational Medicine and Bioinformatics, University of Michigan, Ann Arbor, MI USA; 65 Chan Zuckerberg Biohub, San Francisco, CA USA; 66 Program in Neuroscience, Department of Neurobiology and Behavior, Stony Brook University, Stony Brook, NY USA; 67 Britton Chance Center for Biomedical Photonics, Wuhan National Laboratory for Optoelectronics, MoE Key Laboratory for Biomedical Photonics, Huazhong University of Science and Technology, Wuhan, Hubei China; 68 HUST-Suzhou Institute for Brainsmatics, Suzhou, Jiangsu China; 69 School of Biomedical Engineering, Hainan University, Haikou, Hainan China; 70 SEU-ALLEN Joint Center, Institute for Brain and Intelligence, Southeast University, Nanjing, Jiangsu China; 71 School of Optometry and Ophthalmology, Wenzhou Medical University, Wenzhou, Zhejiang China; 72 Key Laboratory of Intelligent Computation & Signal Processing, Ministry of Education, Anhui University, Hefei, Anhui China; 73 Anhui University, Hefei, Anhui China; 74 Shanghai University, Shanghai, China; 75 School of Computer Engineering and Science, Shanghai University, Shanghai, China; 76 Stanford University, School of Medicine, Stanford, CA USA; 77 Department of Pathology, University of California San Francisco, San Francisco, CA USA; 78 Department of Cell and Molecular Biology, Karolinska Institutet, Stockholm, Sweden; 79 Washington National Primate Research Center, University of Washington, Seattle, WA USA; 80 Jan and Dan Ducan Neurological research Institute, Houston, TX USA; 81 Department of Neurological Surgery, University of Washington School of Medicine, Seattle, WA USA; 82 Regional Epilepsy Center at Harborview Medical Center, Seattle, WA USA; 83 Innovative Genomics Institute, University of California Berkeley, Berkeley, CA USA; 84 Cancer Research Laboratory, University of California Berkeley, Berkeley, CA USA; 85 Semel Institute & Department of Psychiatry and Biobehavioral Science, University of California Los Angeles, Los Angeles, CA USA; 86 David Geffen School of Medicine, University of California Los Angeles, Los Angeles, CA USA; 87 Department of Psychiatry, University of Maryland School of Medicine, Baltimore, MD USA; 88 Center for Bioinformatics and Computational Biology, University of Maryland College Park, College Park, MD USA; 89 Department Neurology & Department Neuroscience, Johns Hopkins School of Medicine, Baltimore, MD USA; 90 CatalystNeuro, Benicia, CA USA; 91 Kitware Inc., Clifton Park, NY USA; 92 Data Sciences Platform, Broad Institute of MIT and Harvard, Cambridge, MA USA; 93 Center for Integrative Connectomics, USC Mark and Mary Stevens Neuroimaging and Informatics Institute, Department of Neurology, Zilkha Neurogenetic Institute, Keck School of Medicine at USC, University of Southern California, Los Angeles, CA USA; 94 Present Address: National Institute of Neurological Disorders and Stroke, National Institutes of Health, Bethesda, MD USA; 95 Present Address: Cajal Neuroscience, Seattle, WA USA; 96 Present Address: Genentech, South San Francisco, CA USA; 97 Present Address: McDonnell Genome Institute, Washington University School of Medicine, St Louis, MO USA; 98 Present Address: Department of Human Genetics, University of California Los Angeles, Los Angeles, CA USA; 99 Present Address: Center for Motor Control and Disease, Key Laboratory of Brain Functional Genomics, East China Normal University, Shanghai, China; 100 Present Address: NYU-ECNU Institute of Brain and Cognitive Science, New York University Shanghai, Shanghai, China; 101 Present Address: Department of Neurosurgery, Stanford University School of Medicine, Stanford, CA USA

**Keywords:** Cellular neuroscience, Molecular neuroscience, Motor cortex, Neural circuits

## Abstract

Here we report the generation of a multimodal cell census and atlas of the mammalian primary motor cortex as the initial product of the BRAIN Initiative Cell Census Network (BICCN). This was achieved by coordinated large-scale analyses of single-cell transcriptomes, chromatin accessibility, DNA methylomes, spatially resolved single-cell transcriptomes, morphological and electrophysiological properties and cellular resolution input–output mapping, integrated through cross-modal computational analysis. Our results advance the collective knowledge and understanding of brain cell-type organization^1–5^. First, our study reveals a unified molecular genetic landscape of cortical cell types that integrates their transcriptome, open chromatin and DNA methylation maps. Second, cross-species analysis achieves a consensus taxonomy of transcriptomic types and their hierarchical organization that is conserved from mouse to marmoset and human. Third, in situ single-cell transcriptomics provides a spatially resolved cell-type atlas of the motor cortex. Fourth, cross-modal analysis provides compelling evidence for the transcriptomic, epigenomic and gene regulatory basis of neuronal phenotypes such as their physiological and anatomical properties, demonstrating the biological validity and genomic underpinning of neuron types. We further present an extensive genetic toolset for targeting glutamatergic neuron types towards linking their molecular and developmental identity to their circuit function. Together, our results establish a unifying and mechanistic framework of neuronal cell-type organization that integrates multi-layered molecular genetic and spatial information with multi-faceted phenotypic properties.

## Abstract (web summary)

The BRAIN Initiative Cell Census Network has constructed a multimodal cell census and atlas of the mammalian primary motor cortex in a landmark effort towards understanding brain cell-type diversity, neural circuit organization and brain function.

## Main

Unique among body organs, the human brain is a vast network of information processing units, comprising billions of neurons interconnected through trillions of synapses. Diverse neuronal and non-neuronal cells display a wide range of molecular, anatomical, and physiological properties that together shape the network dynamics and computations underlying mental activities and behaviour. Brain networks self-assemble during development, leveraging genomic information shaped by evolution to build a set of stereotyped network scaffolds that are largely identical among individuals; life experiences then customize neural circuits in each individual. An essential step towards understanding the architecture, development, function and diseases of the brain is to discover and map its constituent elements of neurons and other cell types.

The notion of a ‘neuron type’, with similar properties among its members, as the basic unit of brain circuits has been an important concept for over a century; however, rigorous and quantitative definitions have remained surprisingly elusive^1–5^. Neurons are remarkably complex and heterogeneous, both locally and in their long-range axonal projections, which can span the entire brain and connect to many target regions. Many conventional techniques analyse one neuron at a time, and often study only one or two cellular phenotypes in an incomplete way (for example, missing axonal arbours in distant targets). As a result, despite major advances in past decades, phenotypic analyses of neuron types have remained severely limited in resolution, robustness, comprehensiveness and throughput. Complexities in the relationship between different cellular phenotypes (multi-modal correspondence) have fuelled long-standing debates on neuronal classification^6^.

Single-cell genomics technologies provide unprecedented resolution and throughput to measure the transcriptomic and epigenomic profiles of individual cells and have rapidly influenced many areas of biology including neuroscience, promising to catalyse a transformation from phenotypic description and classification to a mechanistic and explanatory molecular genetic framework for the cellular basis of brain organization. The application of single-cell RNA sequencing (scRNA-seq) to the neocortex and other brain regions has revealed a complex but tractable hierarchical organization of transcriptomic cell types that are consistent overall with knowledge from decades of anatomical, physiological and developmental studies but with an unmatched level of granularity^7–11^. Similarly, single-cell DNA methylation and chromatin accessibility studies have begun to reveal cell-type-specific genome-wide epigenetic landscapes and gene regulatory networks in the brain^12–15^. Notably, the scalability and high information content of these methods enable comprehensive quantitative analysis and classification of all cell types, which are readily applicable to brain tissues across species and provide a quantitative means of comparative analysis^16,17^.

Other recent technological advances provide the resolution and throughput to analyse whole-brain neuronal morphology and comprehensive projection mapping^18,19^. Imaging-based single-cell transcriptomics and its combination with functional imaging, and integration of electrophysiology and single-cell sequencing, enable mapping of the spatial organization and key phenotypic properties of molecularly defined cell types^20–24^. Finally, molecular classification of cell types enables genetic access to specific cell types using transgenic mice^25–27^ and, more recently, enhancer-based viral vectors^28–32^. All of these methods have been applied to brain tissues in independent studies, but not yet in a coordinated fashion to establish how different modalities correspond with one another, and whether a molecular genetic framework is explanatory for other functionally important cellular phenotypes.

The overarching goal of the BRAIN Initiative Cell Census Network (BICCN) is to leverage these technologies to generate an open-access reference brain cell atlas that integrates molecular, spatial, morphological, connectional and functional data for describing cell types in mouse, human and non-human primate^33^. A key concept is the Brain Cell Census, similar conceptually to a population census, that defines the constituent neuronal and non-neuronal cell types and their proportions, spatial distributions and defining phenotypic characteristics. This cell-type classification, organized as a taxonomy, should aim for consensus across modalities and across mammalian species for conserved types. Beyond the cell census, a Brain Cell Atlas would be embedded in a 3D common coordinate framework (CCF) of the brain^34^, in which the precise location and distribution of all cell types and their multi-modal features are registered and displayed. This spatial framework facilitates integration, interpretation and navigation of various types of information for understanding brain network organization and function.

**Extended Data Fig. 1:**
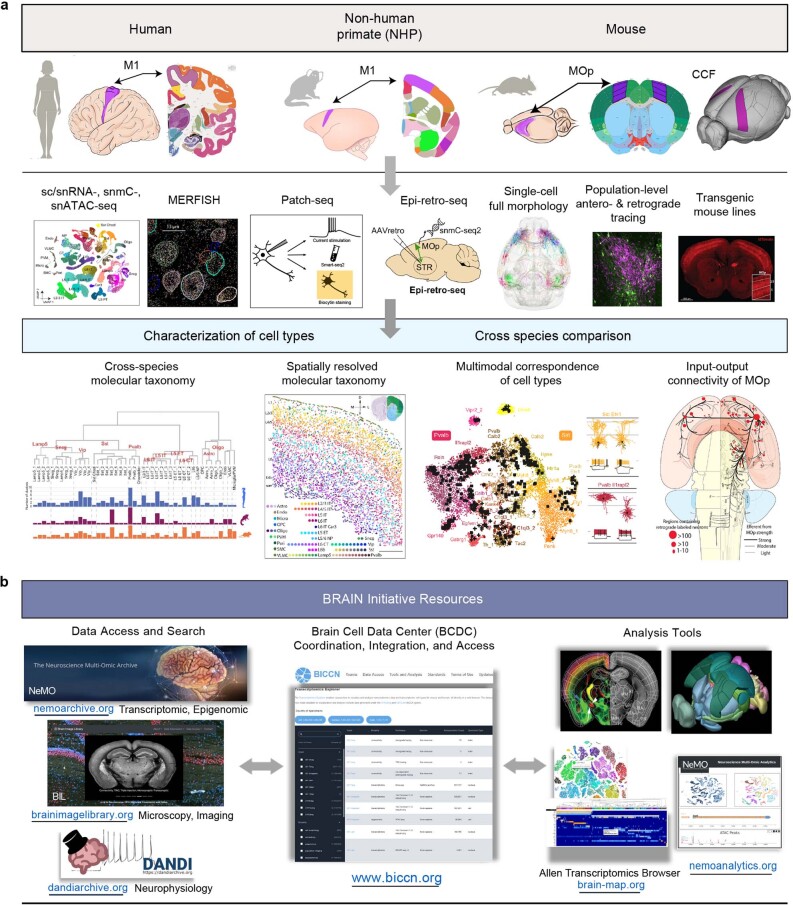
Summary of experimental and computational approaches taken and community resources generated by BICCN. **a**, Comprehensive characterization of cell types in the primary motor cortex (MOp or M1) of three mammalian species using multiple approaches spanning molecular, genetic, physiological and anatomical domains. Integration of these datasets leads to a cohesive multimodal description of cell types in the mouse MOp and a cross-species molecular taxonomy of MOp cell types. **b**, The multimodal datasets are organized by the Brain Cell Data Center (BCDC), archived in the Neuroscience Multi-omic (NeMO) Archive (for molecular datasets), Brain Image Library (BIL, for imaging datasets) and Distributed Archive for Neurophysiology Data Integration (DANDI, for electrophysiology data), and made publicly available through the BICCN web portal www.biccn.org and resource page DOI: 10.5281/zenodo.4726182. Human and mouse icons and brains are credited to Anna Hupalowska at Broad Institute. Marmoset icon and brain are modified from unrestricted use purchase from Shutterstock. Allen mouse CCF, BCDC and transcriptomics browser images are reproduced with permission from Allen Institute. Mouse brain panel in Epi-retro-seq is adapted from https://commons.wikimedia.org/wiki/File:Mouse_brain_sagittal.svg (public domain). DANDI artwork is licensed under CC-BY-3.0 from https://github.com/dandi/artwork.

Here we present the cell census and atlas of cell types in the primary motor cortex (MOp, referred to as M1 in primates) of mouse, marmoset and human (Extended Data Fig. 1, Extended Data Table 1). MOp is important in the control of complex movement and is well conserved across species, with a rich history of anatomical, physiological and functional studies to aid interpretation of this cell-type information^35,36^. We describe a synthesis of eleven companion studies through a coordinated multi-laboratory effort. In these studies, we derive a cross-species consensus molecular taxonomy of cell types using scRNA-seq or single-nucleus RNA sequencing (snRNA-seq), DNA methylation and chromatin accessibility data^37–40^. In mouse, we map the spatial cellular organization by multiplexed error-robust fluorescence in situ hybridization (MERFISH)^41^, characterize morphological and electrophysiological properties by multimodal profiling using patch clamp recording, biocytin staining and scRNA-seq (Patch-seq)^42,43^, describe the cellular input–output wiring diagrams by anterograde and retrograde tracing^44^, identify glutamatergic neuron axon projection patterns by Epi-retro-seq^45^, Retro-MERFISH^41^ and single-neuron complete morphology reconstruction^46^, and describe transgenic driver lines targeting glutamatergic cell types on the basis of marker genes and lineages^47^. Finally, we integrate this information into a cohesive description of cell types in MOp. These datasets are organized by the BRAIN Cell Data Center (BCDC) and made public through the BICCN web portal (https://www.biccn.org). Key concepts and terms are described in Extended Data Table 2, including anatomical terms for input and output brain regions for MOp, and hierarchical cell class, subclass and type definitions.

Major findings:

- Combined single-cell transcriptomic and epigenomic analysis reveals a unified molecular genetic landscape of cortical cell types that integrates gene expression, chromatin state and DNA methylation.
- A combination of single-cell ‘omics, MERFISH-based spatially resolved single-cell transcriptomics and Patch-seq generates a census of cell types, including their proportions and spatial distribution across cortical layers and sublayers.
- Comparative analysis of mouse, marmoset and human transcriptomic types describes a conserved cross-species taxonomy of cortical cell types with hierarchical organization that reflects developmental origins; the transcriptional similarity of cell type granularity across species varies as a function of evolutionary distance.
- We observed highly conserved transcriptomic and epigenomic signatures of cell identity across species, as well as a large set of species-enriched cell-type gene expression profiles that suggests a high degree of evolutionary specialization.
- Correspondence among molecular, anatomical and physiological datasets reinforces the transcriptomic classification of neuronal subclasses and distinctive types, demonstrating their biological validity and genomic underpinnings, and also reveals continuously varying properties along these axes for some neuronal subclasses and types.
- Anatomical studies yield a cellular-resolution wiring diagram of mouse MOp anchored on major transcriptome-defined projection types, including input–output connectivity at the subpopulation level and output pathways at a genetically defined single-cell level.
- Long-range axon projection patterns of individual glutamatergic excitatory neurons exhibit a complex and diverse range of relationships with transcriptomic and epigenetic types (between one-to-one and many-to-many), suggesting another level of regulation in defining single-cell connectional specificity.
- Cell-type transcriptional and epigenetic signatures guide the generation of genetic tools for targeting glutamatergic pyramidal neuron types and fate mapping their progenitor types.
- Multi-site coordination within BICCN and data archives enabled a high degree of standardization, computational integration and creation of open data resources for community dissemination of data, tools and knowledge.

## Results

### Molecular definition of cell types in MOp

**Fig. 1:**
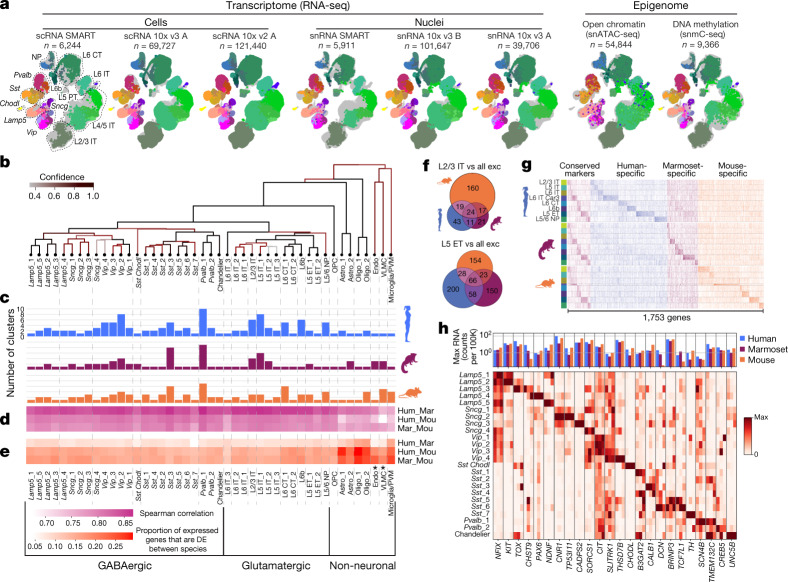
MOp consensus cell-type taxonomy. **a**, Integrated transcriptomic and epigenomic datasets using SCF show consistent molecular cell-type signatures as revealed by a low-dimensional embedding in mouse MOp. Each UMAP plot represents one dataset. Colours indicate cell subclasses. **b**, Dendrogram of integrated human (hum), marmoset (mar) and mouse (mou) cell types based on snRNA-seq datasets (10x v3). Branch colour denotes confidence after 10,000 bootstrap iterations. **c**, Number of within-species clusters included in each cross-species cluster. **d**, **e**, For each cross-species cluster, correlations (**d**) and differentially expressed genes (Wald two-sided test, adjusted *P*-value < 0.05, fold-change > 4) (**e**) between pairs of species. Asterisks denote non-neuronal populations that were under-sampled in human. DE, differentially expressed. **f**, Venn diagrams of shared differentially expressed genes between species for L2/3 IT and L5 ET subclasses. **g**, Conserved and species-enriched differentially expressed genes for all glutamatergic subclasses shown in an expression heat map. **h**, Conserved markers of GABAergic neuron types across three species. Data may be viewed at NeMO Analytics. Marmoset silhouette is adapted from www.phylopic.org (public domain). Exc, excitatory; max, maximum; astro, astrocyte; endo, endothelial cell; oligo, oligodencrocyte; OPC, oligodendrocyte progenitor cell; PVM, perivascular macrophage.

A mouse MOp molecular taxonomy was derived from seven scRNA-seq and snRNA-seq (sc/snRNA-seq) datasets and single-nucleus methylcytosine sequencing (snmC-seq2) and single-nucleus assay for transposase-accessible chromatin using sequencing (snATAC-seq) datasets^37^. The combined sc/snRNA-seq datasets contained a large number of cells profiled using both droplet-based and deep full-length sequencing methods (Extended Data Table 1), resulting in a consensus transcriptomic taxonomy with the greatest resolution compared with other data types, including 90 neuronal and 116 total clusters or transcriptomic types (t-types)^37^. We used this mouse MOp transcriptomic taxonomy as the anchor for comparison and cross-correlation of cell-type classification results across all data types. We further applied two computational approaches, SingleCellFusion (SCF) and LIGER, to combine the transcriptomic and epigenomic datasets and derive an integrated molecular taxonomy consisting of 56 neuronal cell types (corresponding to the 90 transcriptomic neuronal types)^37^ (Fig. 1a). This integrated taxonomy linked RNA transcripts with epigenomic marks identifying potential cell-type-specific *cis*-regulatory elements (CREs) and transcriptional regulatory networks. Similarly, we established M1 cell-type taxonomies for human (127 t-types) and marmoset (94 t-types) by unsupervised clustering of snRNA-seq data, followed by integration with epigenomic datasets^38^.

**Extended Data Fig. 2:**
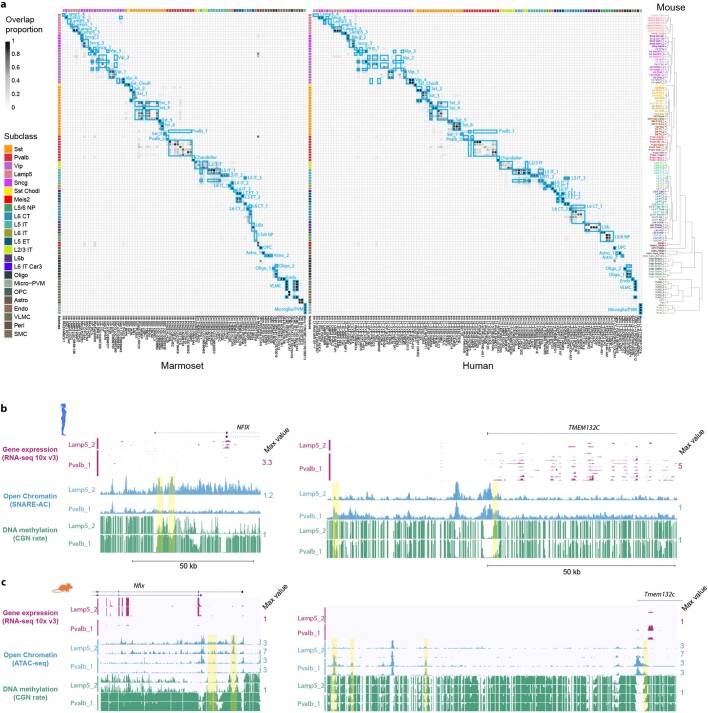
MOp consensus cell type taxonomy. **a**, Cluster overlap heatmap showing the proportion of nuclei in each pair of species clusters that are mixed in the cross-species integrated space. Cross-species consensus clusters are indicated by labeled blue boxes. Mouse clusters (rows) are ordered by the mouse MOp transcriptomic taxonomy dendrogram^37^. Marmoset (left columns) and human (right columns) transcriptomic clusters^38^ are ordered to align with mouse clusters. Color bars at top and left indicate subclasses of within-species clusters. **b-c**, Genome browser view showing transcriptomic and epigenetic signatures for gene markers of *Lamp5*_2 (*NFIX*) and *Pvalb*_1 (*TMEM132C*) GABAergic neurons in human (b) and mouse (c). Yellow bars highlight sites of open chromatin and DNA hypomethylation in the cell type with corresponding marker expression.

To establish a consensus classification of MOp and M1 cell types among mouse, human and marmoset, we integrated snRNA-seq datasets across species and identified 45 conserved t-types, including 24 GABAergic (γ-aminobutyric acid-producing), 13 glutamatergic and 8 non-neuronal types (Extended Data Fig. 2a). The similarity between types was represented as a consensus taxonomy, with branch robustness quantified by using different subsets of genes with variable expression (Fig. 1b). These types were grouped into broader subclasses on the basis of shared developmental origins for GABAergic inhibitory neurons (that is, three caudal ganglionic eminence (CGE)-derived subclasses (*Lamp5*, *Sncg* and *Vip*) and three medial ganglionic eminence (MGE)-derived subclasses (*Sst Chodl*, *Sst* and *Pvalb*)), layer and projection pattern in mouse for glutamatergic excitatory neurons (that is, intratelencephalic (IT), extratelencephalic (ET), corticothalamic (CT), near-projecting (NP) and layer 6b (L6b)), and non-neuronal functional subclasses (for example, oligodendrocytes and astrocytes) (Extended Data Table 2). Note that the layer 5 extratelencephalic (L5 ET) neurons have been called pyramidal tract (PT) or subcerebral projection neurons (SCPN)^48,49^; here we use the name L5 ET to be more accurate across cortical areas and species (Methods).

The resolution of this cross-species consensus taxonomy was lower than that derived from each species alone, owing to variation in gene expression across species. The degree of species alignments varied across consensus types (Fig. 1c); some types could be aligned one-to-one (for example, *Lamp5*_1 and L6 IT_3), whereas others aligned several-to-several (for example, *Pvalb*_1, L2/3 IT and L5 IT_1). This may reflect over- or under-clustering, limitations in aligning highly similar cell types, or species-specific expansion of cell-type diversity.

We expected that cell types from more recent common ancestors would share more similar gene expression profiles. Indeed, transcriptomic profiles of consensus cell types were more correlated between human and marmoset, and had 25–50% fewer differentially expressed genes than between primate and mouse (Fig. 1d, e). The one exception was the vascular leptomeningeal cell (VLMC) type, which had greater Spearman correlations of overall gene expression (Fig. 1d) between marmoset and mouse. However, this probably reflects that rare non-neuronal cells in human (*n* = 40 nuclei) were under-sampled compared with marmoset (*n* = 463) and mouse (*n* = 2,329), and average expression was not adequately estimated^38^.

Glutamatergic subclasses expressed 50–450 marker genes and, unexpectedly, the majority of markers were species-enriched (Fig. 1f, g). This evolutionary divergence of marker gene expression may reflect species adaptations or relaxed constraints on genes that can be substituted with others for related cellular functions. Glutamatergic subclasses also had a core set of 5–65 markers that were conserved across all three species (Fig. 1g); these genes are candidates for conserved cell identity and function, and are useful for consistent labelling across species. GABAergic subclasses expressed 50–325 markers in each species, and 18–55 markers were conserved. At a finer level, GABAergic consensus types also expressed conserved markers with similar expression levels across species and relatively type-specific expression (Fig. 1h). Some marker genes also showed evidence for cell-type-specific enhancers located in regions of open chromatin and DNA hypomethylation in both human and mouse (Extended Data Fig. 2b, c).

### Spatially resolved cell atlas of mouse MOp

We used MERFISH, a single-cell transcriptome imaging method^50,51^, to identify cell types in situ and map their spatial organization. We selected a panel of 258 genes (254 of which passed quality control) on the basis of prior knowledge of marker genes for major cortical cell types and genes identified using sc/snRNA-seq data, and we imaged approximately 300,000 individual cells across MOp and adjacent areas^41^.

**Fig. 2:**
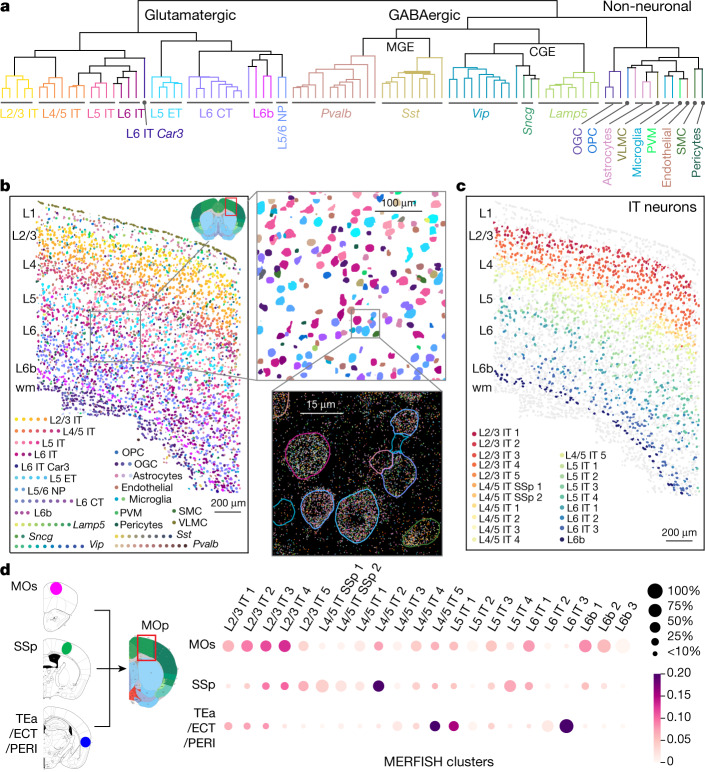
In situ cell-type identification, spatial mapping and projection mapping of individual cells in MOp by MERFISH. **a**, Dendrogram showing the hierarchical relationship among the subclasses and clusters in the mouse MOp identified by MERFISH, coloured by the subclass each cluster belongs to. **b**, Left, spatial map of cell clusters identified in a coronal slice (bregma ~+0.9), with cells coloured by their cluster identity as shown in the colour index. Top right, zoomed-in map of the boxed region of the left panel. Bottom right, spatial localization of individual RNA molecules in the boxed region of the top right panel, coloured by their gene identity. The segmented cell boundaries are coloured according to the cell clusters they belong to. **c**, IT neurons in the same coronal slice as shown in **b**. The IT neurons are coloured by their cluster identity, as shown in the colour index, together with L6b cells in dark blue to mark the bottom border of the cortex. All other cells are shown in grey. **d**, Projection patterns of the MOp neurons into three other regions of the brain, MOs, SSp and TEa–ECT–PERI. Left, CTb was used as retrograde tracer and injected into these three regions. The CTb signals and the MERFISH gene panel were imaged in MOp to determine both the cluster identities and projection targets of individual cells. Projection of MOp neurons to the target regions are displayed as a dot plot, where the size of the dot represents the fraction of cells projecting to each indicated target among all CTb-positive, single-projecting cells in a cluster, and the colour represents the fraction of cells a target received from each indicated cluster. Data may be viewed at NeMO Analytics. OGC, oligodendrocyte; SMC, smooth muscle cell.

Clustering analysis of the MERFISH-derived single-cell expression profiles resulted in a total of 95 cell clusters in MOp (42 GABAergic, 39 glutamatergic and 14 non-neuronal) (Fig. 2a), which showed excellent, essentially one-to-one correspondence to the consensus sc/snRNA-seq taxonomy at subclass level (for example, glutamatergic IT, ET, NP, CT and L6b subclasses, and GABAergic *Lamp5*, *Sncg*, *Vip*, *Sst* and *Pvalb* subclasses) and good correspondence at cluster level^41^.

Spatial distribution of the MERFISH clusters showed a complex, laminar pattern in MOp (Fig. 2b). Many glutamatergic clusters showed narrow distributions along cortical depth that subdivided individual cortical layers, although frequently without discrete boundaries^41^. Notably, IT cells, the largest branch of neurons in the MOp, formed a largely continuous gradient of cells with correlated gradual changes between their expression profiles and their cortical depths^41^ (Fig. 2c). Many GABAergic clusters also showed laminar distribution, preferentially residing within one or two layers^41^. Among the non-neuronal cell clusters, VLMCs formed the outermost layer of cells of the cortex, whereas mature oligodendrocytes and some astrocytes were enriched in white matter. Other subclasses of non-neuronal cells were largely dispersed across all layers. MERFISH analysis also revealed interesting spatial distribution of cell types along the medial–lateral and anterior–posterior axes^41^. Overall, the neuronal and non-neuronal cell clusters in MOp form a complex spatial organization refining traditionally defined cortical layers.

Integration of retrograde tracing with MERFISH (Retro-MERFISH) identified projection targets of different neuron types in the MOp^41^ (Fig. 2d). Retrograde tracers were injected into secondary motor cortex (MOs), primary somatosensory cortex (SSp), and temporal association (TEa) and neighbouring ectorhinal (ECT) and perirhinal (PERI) areas, and retrograde labels were imaged together with the MERFISH gene panel in the MOp (approximately 190,000 cells were imaged). Each of the three target regions received inputs from multiple cell clusters in the MOp, primarily from IT cells; each IT cluster projected to multiple regions, with each region receiving input from a different composition of IT clusters^41^ (Fig. 2d). Overall, projections of MOp neurons do not follow a simple ‘one cell type to one target region’ pattern, but rather form a complex multiple-to-multiple network.

### Multimodal analysis of cell types with Patch-seq

**Fig. 3:**
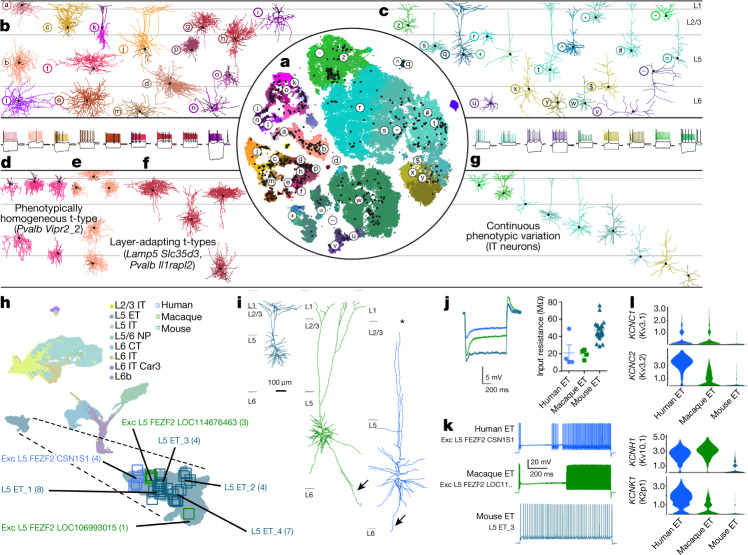
Correspondence between transcriptomic and morpho-electrical properties of mouse MOp neurons by Patch-seq, and cross-species comparison of L5 ET neurons. **a**, *t*-Distributed stochastic neighbor embedding (*t*-SNE) of the scRNA-seq 10x v2 dataset with the superimposed Patch-seq neurons^71^ (black dots). **b**, **c**, Examples of GABAergic interneuron (**b**) and glutamatergic excitatory neuron (**c**) morphologies and electrophysiological recordings. Letters and symbols refer to cells marked in **a**. Three voltage traces are shown in each cell: the hyperpolarization trace obtained with the smallest current stimulation, the first depolarization trace that elicited at least one action potential, and the depolarization trace showing maximal firing rate. Stimulation length, 600 ms. **d**, Example of a phenotypically homogeneous t-type (*Pvalb Vipr2*_2, chandelier neurons). **e**, **f**, Two examples of t-types showing layer-adapting morphologies: *Lamp5 Slc35d3*, neurogliaform cells (**e**) and *Pvalb Il1rapl2*, fast-spiking basket cells (**f**). **g**, Example of a transcriptomic subclass (excitatory IT neurons) that shows continuous within-subclass co-variation between distances in transcriptomic space and morphological space, as seen in similar colour ordering in **a** (right) and **g**. **h**, UMAP visualization of cross-species integration of snRNA-seq data for glutamatergic neurons isolated from mouse, macaque and human, with colours corresponding to cell subclass. Patch-seq samples mapping to various ET neuron types are denoted by squares, colour-coded by species. **i**, Dendritic reconstructions of L5 ET neurons. The human and macaque neurons display classical Betz cell features including taproot dendrites (arrows). Note that the human neuron is truncated (asterisk) before reaching the pial surface. **j**, Voltage response of mouse, macaque and human ET neurons to a 1 s, −300 pA current injection (left) and input resistance (mean ± s.e.m.; macaque *n* = 4, human *n* = 4, mouse *n* = 22) (right). False-discovery rate (FDR)-corrected two-sided Wilcoxon ranked-sum test (human versus mouse *W* = 12, *P* = 0.31, *d* = 2.09; human versus macaque *W* = 5, *P* = 0.49, *d* = 0.08; macaque versus mouse *W* = 0, *P* = 0.0004, *D* = 2.5). **k**, Example spike trains in response to a 10-s suprathreshold current injection. **l**, Violin plots of enriched potassium channel gene expression in human, macaque and mouse L5 ET neurons. Data may be viewed at NeMO Analytics.

We used Patch-seq to characterize the electrophysiological and morphological phenotypes and laminar location of t-types. We patched more than 1,300 neurons in MOp of adult mice, recorded their electrophysiological responses to a set of current steps, filled them with biocytin to recover their morphologies (around 50% of cells) and obtained their transcriptomes using Smart-seq2 sequencing^42^. We mapped these cells to the mouse MOp transcriptomic taxonomy^37^ (Fig. 1a). Cells were assigned to 77 t-types (Fig. 3a), thereby characterizing the morpho-electric phenotypes of most glutamatergic and GABAergic t-types (examples in Fig. 3b, c).

We found that morpho-electric phenotypes were largely determined by transcriptomic subclasses, with different subclasses having distinct phenotypes. For example, *Sst* interneurons were often characterized by large membrane time constants, pronounced hyperpolarization sag, and rebound firing after stimulation offset. However, within each subclass, there was substantial variation in morpho-electric properties between t-types. This variation was not random but organized such that transcriptomically similar t-types had more similar morpho-electric properties than distant t-types. For example, excitatory t-types from the IT subclasses with more similar transcriptomes were also located at adjacent cortical depths, suggesting that distance in t-space co-varied with anatomical distance^42^, even within a layer (Fig. 3g), in line with the above MERFISH results (Fig. 2c). Similarly, electrophysiological properties of *Sst* interneurons varied continuously across the transcriptomic landscape^42^. Thus, within major transcriptomic subclasses, morpho-electric phenotypes and/or soma depth frequently varied smoothly across neighbouring t-types, indicating that transcriptomic neighbourhood relationships in many cases corresponded to similarities in other modalities.

At the level of single t-types, some t-types showed layer-adapting morphologies in different layers (Fig. 3e, f) or even considerable within-type morpho-electric variability within a layer. For example, *Vip Mybpc1*_2 neurons had variable rebound firing strength after stimulation offset. Surprisingly few t-types were entirely homogeneous with regard to the measured morpho-electric properties (Fig. 3d).

Patch-seq also enables direct comparison of the morpho-electric properties of homologous cell types across species. Here we analysed the gigantocellular Betz cells found in M1 of primates and large carnivores, which are predicted to be in the L5 ET subclass^38^, as are the mouse corticospinal-projecting L5 ET neurons. We first created a joint embedding of excitatory neurons in mouse, macaque and human, which showed strong homology across all three species for the L5 ET subclass (Fig. 3h). Patch-seq recordings were made from L5 neurons in acute and cultured slice preparations of mouse MOp and macaque M1. We also capitalized on a unique opportunity to record from neurosurgical tissue excised from human premotor cortex—which also contains Betz cells—during surgery to treat epilepsy. To enable visualization of cells in heavily myelinated macaque M1 and human premotor cortex, we used adeno-associated viruses (AAVs) to drive fluorophore expression in glutamatergic neurons in slice culture.

Patch-seq cells in each species that mapped to the L5 ET subclass (Fig. 3h) were all large L5 neurons that sent apical dendrites to the pial surface (Fig. 3i). Macaque and human L5 ET neurons were much larger, with hallmark Betz cell long ‘taproot’ basal dendrites^52^. Subthreshold membrane properties were relatively well conserved across species. For example, L5 ET neurons in all three species had low input resistances, although they were exceptionally low in macaque and human (Fig. 3j). Conversely, suprathreshold properties of macaque and human Betz ET neurons were highly specialized; they responded to prolonged suprathreshold current injections with biphasic firing in which a pause in firing early in the sweep was followed by a marked increase in firing later (Fig. 3k). Intriguingly, several genes encoding ion channels were enriched in macaque and human L5 ET neurons compared with mouse (Fig. 3l), and may contribute to the distinctive primate suprathreshold properties. These results indicate that primate Betz cells are homologous to mouse thick-tufted L5 ET neurons, but display species specializations in their morphology, physiology and gene expression.

### Multimodal correspondence by Epi-retro-seq

**Fig. 4:**
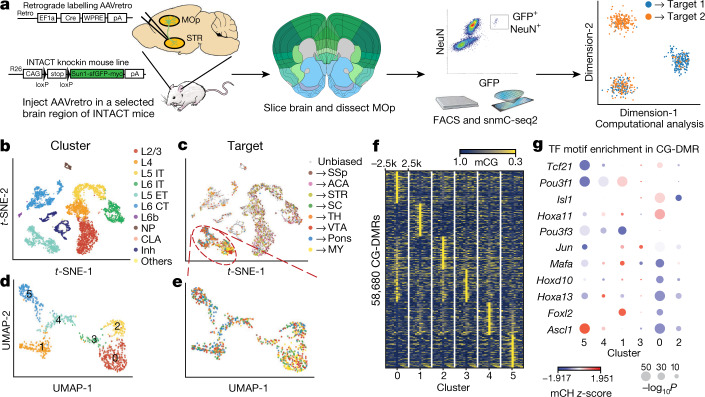
Epi-retro-seq links molecular cell types with distal projection targets. **a**, Workflow of Epi-retro-seq. Mouse brain sagittal panel is adapted from https://commons.wikimedia.org/wiki/File:Mouse_brain_sagittal.svg (public domain). Coronal reference plane is adapted from Allen Reference Atlas with permission. FACS, fluorescence activated cell sorting. **b**, **c**, *t*-SNE of MOp cells profiled by Epi-retro-seq (*n* = 2,115) and unbiased snmC-seq2 (*n* = 4,871) computed with 100-kb-bin-level mCH, coloured by subclasses (**b**) or projection targets (**c**). **d**, **e**, UMAP embedding of L5 ET cells in MOp profiled by Epi-retro-seq (*n* = 848) computed with 100-kb-bin-level mCH, coloured by clusters (**d**) or projection targets (**e**). **f**, mCG levels at CG-DMRs identified between the six clusters and their flanking 2.5-kb regions. Top 100 CG-DMRs in each cluster are shown. **g**, Transcription factor motif enrichment in CG-DMRs in each cluster. Colour represents *z*-scored gene-body mCH level of the transcription factors, and size represents −log_10_ *P* value (computed with Homer, using one-sided binomial tests) of motif enrichment in the CG-DMRs. CLA, claustrum; inh, inhibitory.

To understand molecular diversity among projection neurons, we developed Epi-retro-seq^45^—which combines retrograde tracing and epigenomic profiling—and applied it to mouse MOp neurons projecting to each of the eight selected brain regions receiving inputs from MOp (Fig. 4a). Th- target regions included two cortical areas, SSp and anterior cingulate area (ACA), and six subcortical areas, striatum (STR), thalamus (TH), superior colliculus (SC), ventral tegmental area and substantia nigra (VTA+SN), pons and medulla (MY).

**Extended Data Fig. 3:**
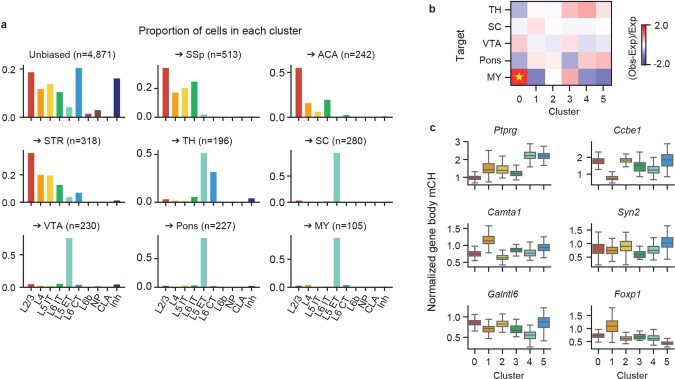
Epi-retro-seq links molecular cell types with distal projection targets. **a**, Distribution across subclasses of neurons from unbiased snmC-seq2 and neurons projecting to each target. **b**, Enrichment of L5 ET neurons projecting to each target in each cluster. * represents FDR < 0.05, Wald test, Benjamini-Hochberg Procedure. **c**, Boxplots of normalized mCH levels at gene bodies of example CH-DMGs in the six clusters. Numbers of cells represented by the boxes are 242, 165, 118, 42, 119, and 162 for the six clusters. The elements of boxplots are defined as: center line, median; box limits, first and third quartiles; whiskers, 1.5× interquartile range.

We obtained methylomes for 2,115 MOp projection neurons. Co-clustering them with MOp neurons collected without enrichment of specific projections, we observed precise agreement among all major cell subclasses (Fig. 4b, c). We observed enrichment of cortico-cortical and cortico-striatal projecting neurons in IT subclasses (L2/3, L4, L5 IT, L6 IT and L6 IT *Car3*), and cortico-subcortical projecting neurons in L5 ET. Many cortico-thalamic projecting neurons were also observed in L6 CT (Extended Data Fig. 3a). Consistent with the specificity of retrograde labelling, quantitative comparisons with unbiased collection of neurons in MOp suggest at least 30-fold (IT) or 200-fold (ET) enrichment of neurons in the expected subclasses (Methods).

Enrichment of L5 ET neurons with Epi-retro-seq (40.2% versus 5.62% in unbiased profiling of MOp using snmC-seq2) enabled investigation of subtypes of L5 ET neurons known to project to multiple subcortical targets in TH, VTA+SN, pons and MY^48^. The 848 L5 ET neurons were segregated into 6 clusters (Fig. 4d, e). MY-projecting neurons showed clear enrichment for L5 ET cluster 0 (Fig. 4e, Extended Data Fig. 3b), in agreement with scRNA-seq data for anterolateral motor cortex (ALM), part of MOs^9,53^. We used gene body non-CG methylation (mCH) levels to integrate the L5 ET Epi-retro-seq cells with the ALM Retro-seq cells and observed enrichment of MY-projecting cells in the same cluster^45^.

The presence of mCH in gene bodies is strongly anti-correlated with gene expression in neurons, whereas promoter-distal differentially CG-methylated regions (CG-DMRs) are reliable markers of regulatory elements such as enhancers^12^. We identified 511 differentially CH-methylated genes (CH-DMGs) and 58,680 CG-DMRs across the L5 ET clusters (Fig. 4f). We also inferred transcription factors that may contribute to defining the cell clusters by identifying enriched transcription factor-binding DNA sequence motifs within CG-DMRs (Fig. 4g). For example, *Ascl1* is a transcription factor whose motif was significantly enriched in the MY-projecting cluster. In addition, 230 hypo-CH-DMGs were identified between the MY-projecting cluster and other projection neurons. One of the most differentially methylated genes is *Ptprg* (Extended Data Fig. 3c), which encodes the receptor tyrosine phosphatase-γ, which interacts with contactin proteins to mediate neural projection development^54^. Thus, these epigenomic mapping data for projection neurons facilitate the understanding of gene regulation in establishing neuronal identity and connectivity.

### Cell-type-targeting tools

Genetic access to specific neural subpopulations and progenitors is necessary for multi-modal analyses to validate t-types, fate-map their developmental trajectories, and study their function in circuit operation^25^. Here we present a genetic toolkit for dissecting and fate-mapping glutamatergic pyramidal neuron (PyN) subpopulations largely on the basis of their developmental genetic programs.

**Fig. 5:**
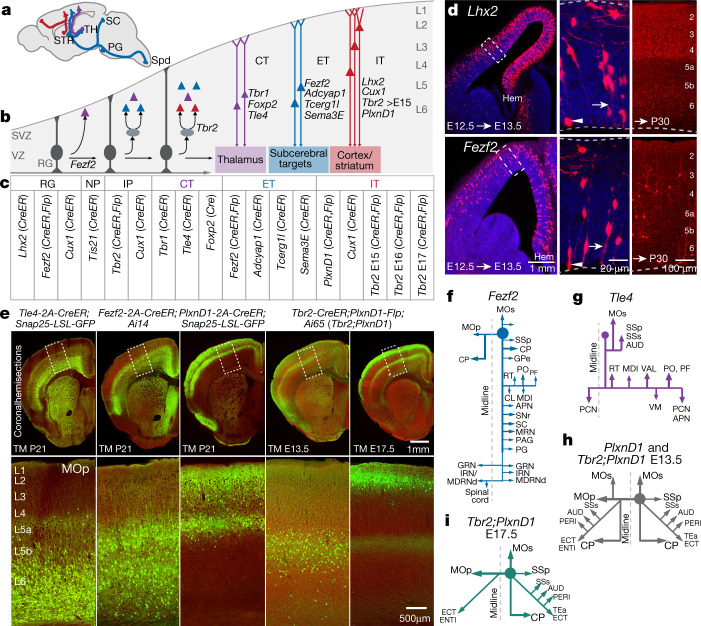
Genetic tools for targeting cortical glutamatergic projection neuron types. **a**, Major PyN projection classes mediating IT (red) and cortical output channels (ET, blue; CT, purple). PG, pontine grey; Spd, spinal cord. **b**, Developmental trajectory from progenitors to mature PyNs. Genes specify lineage and projection types. VZ, ventricular zone; SVZ, subventricular zone. **c**, New gene knockin driver mouse lines targeting RG, neurogenic precursor (NP), IP and broad projection types. **d**, Pulse-chase of E12.5 *Lhx2-2A-CreER;Ai14* (*Lhx2*) (top row) and *Fezf2-2A-CreER;Ai14* (*Fezf2*) (bottom row) embryos for 24 hours densely labelled RGs throughout dorsal neuroepithelium (left). Middle, boxed areas shown left, magnified, showing RG^*Lhx2+*^ and RG^*Fezf2+*^. Long pulse-chase (right) of E12.5 RGs generates PyNs across layers at postnatal day (P)30. Arrows show endfeet and arrowheads show dividing soma. Hem, cortical hemisphere. **e**, Driver–reporter recombination patterns (reporter, pseudo-coloured green; background, red) from five PyN subpopulations defined by *Tle4*, *Fezf2*, *PlxnD1* and *Tbr2;PlxnD1* with tamoxifen (TM) induction times. Combinatorial definition of PyN^*PlxnD1*^ subtypes by lineage, birth time and anatomical location achieved by *Tbr2;PlxnD1* intersection: tamoxifen at E13.5 and at E17.5 labelled different *Tbr2*-expressing IP-derived PyN^*PlxnD1*^ cohorts. Boxed areas in MOp (top row) are shown in the bottom row. **f**–**i**, Main PyN subpopulation projection targets from MOp. Drivers were crossed with mouse reporter lines, *Rosa26-CAG-LSL-Flp* (Cre-dependent) or *Rosa26-CAG-*dual*-(LSL-FSF)-*tTA (Cre-AND-Flp-dependent), and tamoxifen induction was performed to convert transient CreER to constitutive reporter expression for anterograde tracing with Flp- or tTA-dependent AAV vector (AAV8-CAG-fDIO-TVA-EGFP or AAV-TRE-3g-TVA-EGFP). Filled circle shows MOp injection site. For full names of projection target acronyms, see refs. ^34,47^. APN, anterior pretectal nucleus; AUD, auditory cortex; CL, central lateral nucleus; ENTI, entorhinal area, lateral part; GPe, globus pallidus, external segment; GRN, gigantocellular reticular nucleus; IRN, intermediate reticular nucleus; MDl, mediodorsal nucleus, lateral part; MDRNd, medullary reticular nucleus, dorsal part; MRN, midbrain reticular nucleus; PAG, periaqueductal grey; PCN, paracentral nucleus; PF, parafascicular nucleus; RT, reticular nucleus; SNr, substantia nigra, reticular part; SSs, supplemental somatosensory cortex.

Along the lineage progression of neural progenitors during corticogenesis in the embryonic dorsal telencephalon, radial glial progenitors (RGs) generate PyNs either directly or indirectly through intermediate progenitors (IPs)^55^ (Fig. 5a, b). Temporal expression of transcription factors gates sequential developmental decisions to shape hierarchically organized PyN subpopulations^47,56^. The LIM-homeodomain protein LHX2 and zinc-finger transcription factor FEZF2 act at multiple stages of neurogenesis^55,57^, and IPs specifically express the T-box transcription factor *Tbr2* during indirect neurogenesis^58^. We generated temporally inducible *Lhx2-CreER*, *Fezf2-CreER*, *Tbr2-CreER*, *Fezf2-Flp* and *Tbr2-FlpER* driver lines (Fig. 5c) that faithfully recapitulate the spatiotemporal expression of these transcription factors and enable fate-mapping of associated RG and IP pools^47^. For example, *Lhx2-CreER* and *FezF2-CreER* drivers captured embryonic day (E)12.5 RGs in the dorsal neuroepithelium, distributed along a medial-high and lateral-low gradient, consistent with their mRNA expression at this stage^59,60^. These RGs generated PyNs across all cortical layers, suggesting multipotency (Fig. 5d).

We also generated 15 Cre and Flp driver lines targeting PyN subpopulations, including the CT, PT and IT subclasses, and subpopulations within these subclasses (Fig. 5b, c). These driver lines precisely recapitulated endogenous expression patterns, highlighted here with three representative lines (Fig. 5e): L2/3 and L5a for IT-*Plxnd1* (IT^*Plxnd1*^), L5b and L6 for ET-*Fezf2* (ET^*Fezf2*^), L6 for CT-*Tle4* (CT^*Tle4*^). Anterograde projection tracing in MOp of adult animals demonstrated that IT^*Plxnd1*^ projected to multiple ipsilateral and contralateral cortical areas and to STR/caudate putamen (CP); ET^*Fezf2*^ projected robustly to several ipsilateral cortical sites, CP and numerous subcortical targets including TH, MY and corticospinal tract; CT^*Tle4*^ projected specifically to a set of thalamic nuclei^47^ (Fig. 5f–h).

We further developed a combinatorial method to target PyN subtypes on the basis of their lineage, birth order and anatomical features. For example, the PyN^*PlxnD1*^ population localizes to L5a, L3 and L2 and projects to many ipsilateral and contralateral cortical and striatal targets^47^ (Fig. 5e, h). Based on the knowledge that most IT PyNs are generated from IPs^61^, we generated *PlxnD1-Flp;Tbr2-CreER;Ai65* compound mice in which the inducible *Tbr2-CreER* allele was used to birth date IT^*PlxnD1*^. Tamoxifen induction at E13.5 and 17.5 selectively labelled L5a and L2 IT^*PlxnD1*^, respectively, across cortical areas (Fig. 5e). To reveal their projection patterns, we bred the *PlxnD1-Flp;Tbr2-CreER;*dual-tTA mice for tTA-dependent viral tracing in MOp. We found that E13.5-born IT^*PlxnD1*(E13.5)^ neurons resided in L5a and projected ipsilaterally to multiple cortical areas, contralaterally to homotypic and heterotypic areas, and bilaterally to CP (Fig. 5h). By contrast, E17.5-born IT^*PlxnD1*(E17.5)^ neurons resided in L2; although they also projected to ipsilateral cortical and striatal targets, and to homotypic contralateral cortex, they extended minimal projections to heterotypic contralateral cortex and CP (Fig. 5i). Together, this set of PyN driver lines provides much-improved specificity, robustness, reliability and coverage, and demonstrates feasibility to target highly specific PyN subtypes.

### MOp input–output wiring diagram

A comprehensive cellular resolution input–output MOp wiring diagram was generated by combining classic tracers, genetic viral labelling in Cre driver lines and single-neuron reconstructions with high-resolution, brain-wide imaging, precise 3D registration to CCF and computational analyses^44^.

**Fig. 6:**
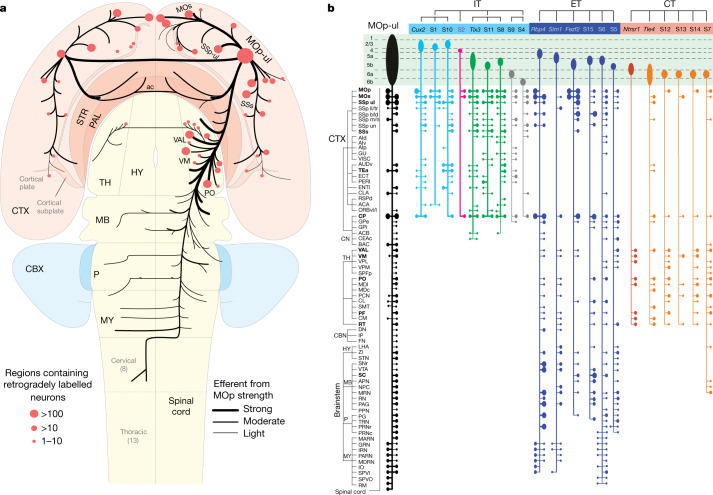
Global wiring diagram and anatomical characterization of MOp-ul neuron types. **a**, Flat map representation of the MOp-ul input–output wiring diagram. Black lines and red dots indicate axonal projections (outputs) and retrograde labelling sources (inputs), respectively, with line thickness and dot sizes representing relative connection strengths. Most MOp-ul projection targets in the cortex and TH also contain input sources, suggesting bi-directional connections. Numbers in parentheses indicate numbers of cervical or thoracic segments in spinal cord. **b**, Projection patterns arising from excitatory cell subclasses, IT, ET and CT, with corresponding Cre line assignment and somatic laminar location, compared with the overall projection pattern from the MOp-ul region (left, black). Along each vertical output pathway, horizontal bars on the right and left sides represent ipsilateral and contralateral collaterals, respectively, with dot sizes indicating the strength of axonal termination in different targets. For full names of projection target acronyms, see refs. ^34,44^. ac, anterior commissure; ACB, nucleus accumbens; AId, v, p, agranular insular cortex, dorsal, ventral, posterior part; AUDv, ventral auditory cortex; BAC, bed nucleus of the anterior commissure; CBN, cerebellar nuclei; CBX, cerebellar cortex; CEAc, central amygdalar nucleus, capsular part; CM, central medial nucleus; CN, cerebral nuclei; CTX, cerebral cortex; DN, dentate nucleus; FN, fastigial nucleus; GPi, globus pallidus, internal segment; GU, gustatory cortex; HY, hypothalamus; IO, inferior olivary complex; IP, interposed nucleus; LHA, lateral hypothalamic area; MARN, magnocellular reticular nucleus; MB, midbrain; MDc, mediodorsal nucleus, central part; MDRN, medullary reticular nucleus; NPC, nucleus of the posterior commissure; ORBvl, l, orbital cortex, ventrolateral, lateral part; P, pons; PAL, pallidum; PARN, parvicellular reticular nucleus; PPN, pedunculopontine nucleus; PRNr, pontine reticular nucleus; PRNc, pontine reticular nucleus, caudal part; RM, nucleus raphe magnus; RN, red nucleus; RSPd, retrosplenial cortex, dorsal part; SMT, submedial nucleus; SPFp, subparafascicular nucleus, parvicellular part; SPVI, spinal nucleus of the trigeminal, interpolar part; SPVO, spinal nucleus of the trigeminal, oral part; SSp-ul, -ll, -tr, -bfd, -m, -n, -un, primary somatosensory cortex upper limb, lower limb, trunk, barrel field, mouth, nose, unassigned; STN, subthalamic nucleus; TRN, tegmental reticular nucleus; VISC, visceral cortex; VPL, ventral posterolateral nucleus; VPM, ventral posteromedial nucleus; ZI, zona incerta.

We first systematically characterized the global inputs and outputs of MOp upper limb (MOp-ul) region using classic anterograde (*Phaseolus vulgaris* leucoagglutinin (PHAL)) and retrograde (cholera toxin b (CTb)) tract tracing^44^ (Fig. 6a). MOp-ul projects to more than 110 grey matter regions and spinal cord, and around 60 structures in the cerebral cortex and TH project back to MOp-ul.

**Extended Data Fig. 4:**
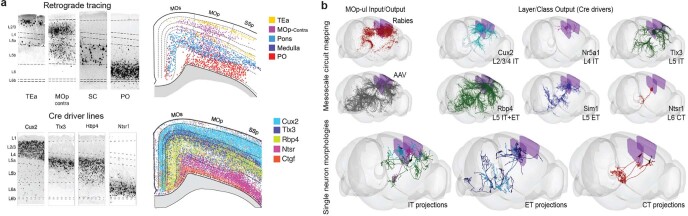
Anatomical characterization of MOp-ul neuron types. **a**, MOp-ul neurons classified by projection targets or transgenic Cre expression. Top, retrograde tracing using CTb revealed layer-specific distributions of MOp-ul neurons with respect to their major projection targets. Representative images (left) show neurons labeled by CTb injections into cortical areas (TEa, contralateral MOp), SC in the midbrain, and PO of the thalamus. Detected cells were pseudo-colored and overlaid onto a schematic coronal section near the center of MOp-ul (right). MOp neurons that project to TEa are distributed in L2 and L5 (yellow), to the contralateral MOp in L2-L6b (purple), to targets in the pons and medulla in L5b (blue), and to thalamus in L6a (red). Bottom, distribution of neurons labeled in transgenic Cre lines was mapped in MOp and across the whole cortex. Images (left) show laminar patterns of Cre+ nuclei in MOp-ul from four driver lines (*Cux2*, *Tlx3*, *Rbp4*, and *Ntsr1*). Detected nuclei from these lines, plus the *Ctgf-Cre* line, were pseudo-colored and overlaid onto a schematic coronal section near the center of MOp-ul (right). Cre+ nuclei are found in L2-4 in *Cux2*; L5a and superficial L5b in *Tlx3*; L5a and L5b in *Rbp4*; L6a in *Ntsr1*, and L6b in *Ctgf*. **b**, 3D views show brain-wide MOp input–output patterns at the population and single cell resolution. Top left, regional MOp inputs and outputs were mapped using retrograde (in red, example showing rabies tracing from the *Tlx3-Cre* line) and anterograde (in black, example showing AAV-EGFP) tracing methods. Top right, whole-brain axonal trajectories from 6 Cre line-defined subpopulations labeled with Cre-dependent AAV tracer injections at the same MOp-ul location. Bottom, individual projection neurons were fully reconstructed following high-resolution whole-brain imaging of sparsely labeled cells. Representative examples of IT, ET, and CT neurons are shown in each panel. The two ET examples represent distinct projection types; medulla (dark blue)- and non-medulla-projecting (light blue). 3D renderings were generated following registration of projection and reconstruction data into CCFv3 using BrainRender^88^.

We generated a fine-grained areal and laminar distribution map of multiple MOp-ul projection neuron populations using retrograde tracing^44^ (Extended Data Fig. 4a). In parallel with these tracer-labelled, projection- and layer-defined cell populations, we characterized the distribution patterns in MOp-ul of neuronal populations labelled in 28 Cre driver lines, including those from different IT (for example, *Cux2*, *Plxnd1* and *Tlx3* driver lines), ET (*Rbp4*, *Sim1* and *Fezf2*) and CT (*Ntsr1* and *Tle4*) subclasses with distinct laminar distributions^47,62^.

Viral tracers were used to systematically examine MOp-ul cell subclass-specific inputs and outputs^44^ (Extended Data Fig. 4b). Neurons projecting to Cre-defined starter cells were labelled using trans-synaptic rabies viral tracers. Projections from MOp were labelled following AAV-GFP injection into wild-type mice, revealing patterns consistent with PHAL tracing (Fig. 6a). Projections from L2/3 IT, L4 IT, L5 IT, L5 ET and L6 CT cells were mapped following injections of Cre-dependent viral tracers into Cre lines selective for these laminar and projection cell subclasses^63^. Most Cre line anterograde tracing experiments revealed a component of the overall output pathway. This result is consistent with labelling from retrograde injections in various thalamic nuclei (posterior complex (PO), ventral anterior-lateral complex (VAL) and ventral medial nucleus (VM)) and cortical areas such as MOs and SSp.

We systematically characterized axonal projections of more than 300 single MOp excitatory neurons, by combining sparse labelling, high-resolution whole-brain imaging, complete axonal reconstruction and quantitative analysis^44,46^, augmented with publicly available single-cell reconstructions from the Janelia Mouselight project^18^. Additional analysis was also conducted using BARseq^44,64^. This analysis revealed a rich diversity of projection patterns within the IT, ET and CT subclasses (Fig. 6b). Individual L6 neurons display several distinct axonal arborization targets that likely contribute to the composite subpopulation output described for the *Ntsr1* and *Tle4* diver lines. Individual IT cells across L2–L6 also generate richly diverse axonal trajectories. Confirming and extending previous reports^53^, we characterized detailed axon projections of the MY-projecting and non-MY-projecting L5 ET neurons, revealing complex axon collaterals in TH and midbrain regions^44,46^.

### Multimodal characterization of L4 IT neurons in MOp

**Fig. 7:**
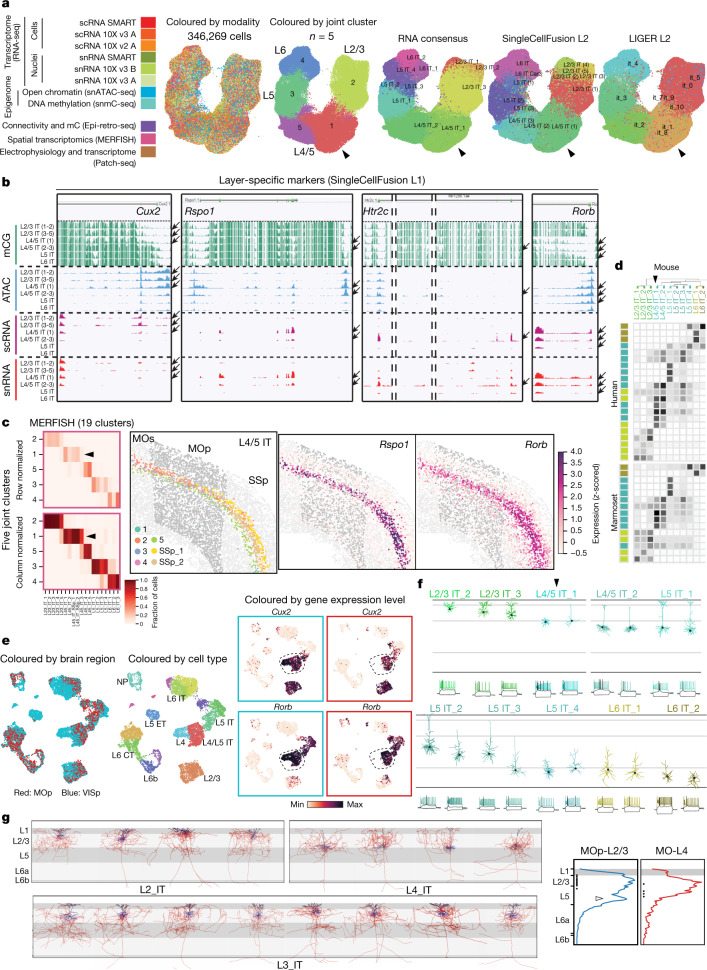
Existence of L4 excitatory neurons in MOp. **a**, UMAP embedding of IT cells from 11 datasets. Cells are coloured by modalities, by cluster identities from the 11-dataset joint clustering, and by cluster identities generated from other consensus clustering^37^. **b**, Genome browser view of layer-specific gene markers—from L2/3 to L5—across IT cell types (SCF L1)^37^. Arrows indicate cell types with correlated transcription and epigenomic signatures of the specific marker gene. **c**, MERFISH IT clusters correspond well with the joint clusters from **a** (confusion matrices, left), and reveal a group of L4 specific clusters (L45_IT) between L2/3 and L5 and marked by genes *Rspo1* and *Rorb* (right). **d**, Correspondence between mouse and human or marmoset transcriptomic IT types. **e**, UMAP embedding of excitatory cells from MOp and VISp. Gene expression levels are log_10_(transcripts per million + 1). **f**, Dendritic morphologies and spiking patterns of mouse Patch-seq cells from L2/3-6 IT types. Arrowheads in **a**, **c**, **d**, **f** indicate the L4/5 IT_1 type. **g**, Left, local dendritic and axonal morphologies of fully reconstructed IT neurons with somas located in L2, L3 and L4. Black, apical dendrites. Blue, basal dendrites. Red, axons. Right, quantitative vertical profiles showing average distribution of local axons along cortical depth for L2/3 or L4 neurons. Dots indicate soma locations and the open arrowhead points to L2/3 neuron axon projections down to L5. Layer marking is approximate owing to the variable thickness of layers in different parts of MOp.

Traditionally MOp has been considered an agranular cortical area, defined by the lack of a cytoarchitectonic layer 4, which usually contains spiny stellate or star pyramid excitatory neurons. However, previous studies have suggested that L4 neurons similar to those typically found in sensory cortical areas are also present in mouse MOp and macaque M1^65,66^. Here we present multimodal evidence to confirm the presence of L4-like neurons in mouse MOp and primate M1 (Fig. 7).

We performed a joint clustering (Methods) and uniform manifold approximation and projection (UMAP) embedding of all IT neurons (excluding the highly distinct L6 IT Car3 cells) from 11 mouse molecular datasets, including 6 sc/snRNA-seq datasets, and the snmC-seq2, snATAC-seq, Epi-retro-seq, MERFISH and Patch-seq data (Fig. 7a). This resulted in five joint clusters, mostly along a continuous variation axis from L2/3 to L4/5 to L5 to L6 in line with the above MERFISH and Patch-seq results. The joint clustering enabled linkage of the cells independently profiled by each individual modality and cross-correlation of these disparate properties. Consequently, we identified epigenomic peaks linked to cluster-specific marker genes—*Cux2* for L2/3 IT and L4/5 IT (1), *Rspo1* for L4/5 IT (1), *Htr2c* for L4/5 IT (2-3), and *Rorb* for L4/5 IT and L5 IT (Fig. 7b, cluster names from SCF). MERFISH data showed that L4/5 IT and L5 IT cells occupied distinct layers in MOp, and the L4/5 IT type expressed *Rspo1* (Fig. 7c), a L4 cell-type marker in sensory cortical areas identified in previous studies^9^. There are fewer *Rspo1*^+^ L4 cells in MOp than in the neighbouring SSp. Transcriptomic IT types from mouse corresponded well with those from human and marmoset at subclass level, whereas substantial ambiguities existed at cluster level (Fig. 7d), probably owing to the gene expression variation between rodents and primates (Fig. 1).

We further compared the L4 cells in mouse MOp with those from mouse primary visual cortex (VISp)^9^ after co-clustering all the SMART-seq glutamatergic transcriptomes from both regions (Fig. 7e). In UMAP, L4/5 IT cells in MOp occupied a subspace of the L4 IT co-cluster defined by the intersection of marker genes *Cux2* and *Rorb*, suggesting that L4 cells in MOp are similar to a subset of L4 cells in VISp, while the L4 cells in VISp have additional diversity and specificity.

L4 IT cells in MOp also exhibited morphological features characteristic of traditionally defined L4 excitatory neurons. In Patch-seq^42^, cells from the L4/5 IT_1 type had no or minimal apical dendrites without tufts in L1, in contrast to cells from the L2/3 IT, L4/5 IT_2 and L5 IT types, which had tufted apical dendrites (Fig. 7f). We obtained complete morphological reconstructions of excitatory neurons with their somas located in L2, L3 or L4 in MOp or MOs from fMOST imaging of *Cux2-CreERT2;Ai166* mice^46^. The reconstructed MOp or MOs neurons with somas in putative L4 (between L2/3 and L5) exhibited two local morphological features typical of L4 neurons from sensory cortices (Fig. 7g). First, the dendrites of the L4 neurons were simple and untufted, whereas those of the L2/3 neurons all had extensive tufts. Second, the local axons of L4 neurons mostly projected upward into L2/3 in addition to collateral projections, whereas the local axons of L2/3 neurons had axon branches projecting downward into L5. These local projection patterns are consistent with the canonical feedforward pathways within a cortical column observed in somatosensory and visual cortices, with the first feedforward step from L4 to L2/3 and the second feedforward step from L2/3 to L5^67^. We also found that the MOp or MOs L4 neurons had intracortical long-range projections similar to the L2/3 neurons^46^ (Fig. 6b).

### Multimodal characterization of L5 ET neurons in MOp

Previous studies showed that in mouse ALM, L5 ET neurons have two transcriptomically distinct projection types that may be involved in different motor control functions: the TH-projecting type in movement planning and the MY-projecting type in movement initiation^53^. Here we demonstrate that L5 ET neurons in mouse MOp also have MY-projecting and non-MY-projecting types, with distinct gene markers, epigenomic elements, laminar distribution, genetic targeting tools and corresponding types in human and marmoset.

**Fig. 8:**
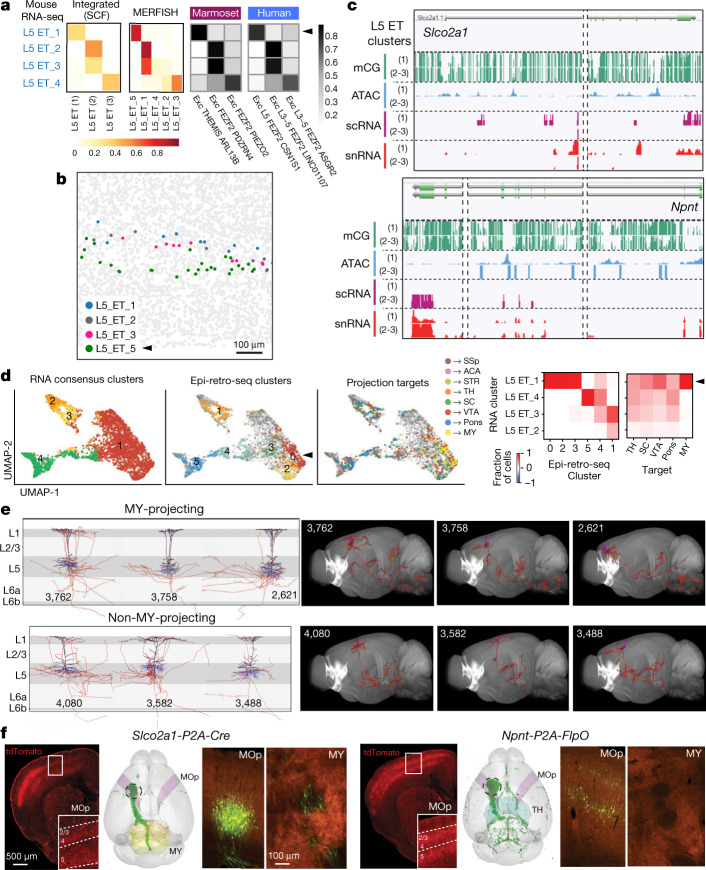
Two distinct L5 ET projection neuron types in MOp. **a**, Confusion matrices between mouse L5 ET RNA-seq clusters and SCF, MERFISH, human or marmoset clusters, with the fraction of cells in each of the other datasets mapped to mouse transcriptomic clusters. **b**, Distribution of MERFISH L5 ET cells in upper or lower L5. L5_ET_4 cells are not found in this section. **c**, Genome browser view of gene markers for the MY-projecting (*Slco2a1*) and non-MY-projecting (*Npnt*) L5 ET neurons. **d**, Left, integration UMAP panels between L5 ET Epi-retro-seq clusters and consensus transcriptomic clusters using the snRNA 10x v3 B dataset with the largest number of L5 ET cells (more than 4,000). Right, confusion matrices (normalized by columns). **e**, Local dendritic and axonal morphologies (left) and brain-wide axon projections (right) of fully reconstructed MY-projecting and non-MY-projecting L5 ET neurons. Black, apical dendrites; blue, basal dendrites; red, axons. Numbers are cell IDs. **f**, Characterization of two L5 ET driver lines. For each line, the first panel shows tdTomato reporter expression from a *Slco2a1-P2A-Cre;Ai14* or *Npnt-P2A-FlpO;Ai65F* mouse. The second panel shows a whole-brain view of the projection pattern from a *Slco2a1-P2A-Cre* mouse injected with AAV-pCAG-FLEX-EGFP-WPRE or a *Npnt-P2A-FlpO* mouse injected with AAV-pCAG-fDIO-mNeonGreen-WPRE (MOp (purple), TH (blue) and MY (yellow); injection sites indicated by the dashed circles). Last two panels show GFP- or mNeonGreen-labelled neurons at the injection site (MOp) and axon fibres in contralateral MY (seen only in *Slco2a1* but not *Npnt*).

Compared with the previous VISp–ALM transcriptomic taxonomy^9^, mouse MOp L5 ET_1 type corresponded to the ALM MY-projecting type, whereas MOp L5 ET_2-4 types corresponded to the ALM TH-projecting types^37^. Here we show that this distinction is consistent across all molecular datasets (Fig. 8a). L5 ET_1 or L5 ET_2-4 types corresponded well with SCF type L5 ET (1) or L5 ET (2-3) and MERFISH cluster L5_ET_5 or L5_ET_1-4, respectively, as well as with different L5 ET types from human and marmoset. The laminar distribution of these two groups was revealed by MERFISH, with L5_ET_1-4 cells intermingled in the upper part of L5 and L5_ET_5 cells located distinctly in lower L5 (Fig. 8b). The two groups were further distinguished by epigenomic peaks associated with specific marker genes, *Slco2a1* for SCF L5 ET (1) type and *Npnt* for SCF L5 ET (2-3) types (Fig. 8c).

Epi-retro-seq revealed more complex long-range projection patterns among the 6 epigenetic L5-ET clusters identified, with MY projection cells predominantly in cluster 0 but also in clusters 2 and 3 (Extended Data Fig. 3b). We co-clustered L5 ET cells from the Epi-retro-seq data and the snRNA-seq 10x v3 B data^37^, and found that the consensus transcriptomic cluster L5 ET_1 corresponded to Epi-retro-seq clusters 0, 2 and 3, whereas transcriptomic clusters L5 ET_2-4 corresponded to Epi-retro-seq clusters 1, 4 and 5, which contain almost no MY-projecting neurons (Fig. 8d).

We identified multiple full-morphology reconstructions of MOp L5 ET neurons from fMOST imaging of *Fezf2-CreER;Ai166* and *Pvalb-T2A-CreERT2;Ai166* transgenic mice, which were clustered into MY-projecting and non-MY projecting morphological types but also exhibited extensive morphological and projectional variability among individual cells^46^ (Fig. 8e), although this was not directly linked to t-types. Both groups of cells had thick-tufted dendrites that were similar to each other (Fig. 8e), consistent with the Patch-seq study^42^.

We used CRISPR–Cas9 gene editing to generate transgenic mice in which Cre or Flp recombinase was targeted to *Slco2a1* or *Npnt*, marker genes for the MY-projecting or non-MY-projecting L5 ET type, respectively (Fig. 8c). Cre- and Flp-dependent tdTomato reporter in *Slco2a1-P2A-Cre;Ai14* and *Npnt-P2A-FlpO;Ai65F* mice labelled cortical L5 neurons, as well as vascular cells in *Slco2a1* mice and L2/3 cells in *Npnt* mice (Fig. 8f). *Slco2a1*-labelled cells occupy a deeper sub-lamina of L5 than those targeted by *Npnt*, consistent with the MERFISH result (Fig. 8b). To test the projection specificity of labelled neurons, we injected AAV vectors encoding a Cre- or Flp-dependent EGFP reporter into L5 in the MOp of these mice. GFP-labelled axon terminals were found in MY of *Slco2a1* but not *Npnt* mice, demonstrating cell-type specificity of these new driver lines (Fig. 8f).

### An integrated synthesis of MOp cell types

**Fig. 9:**
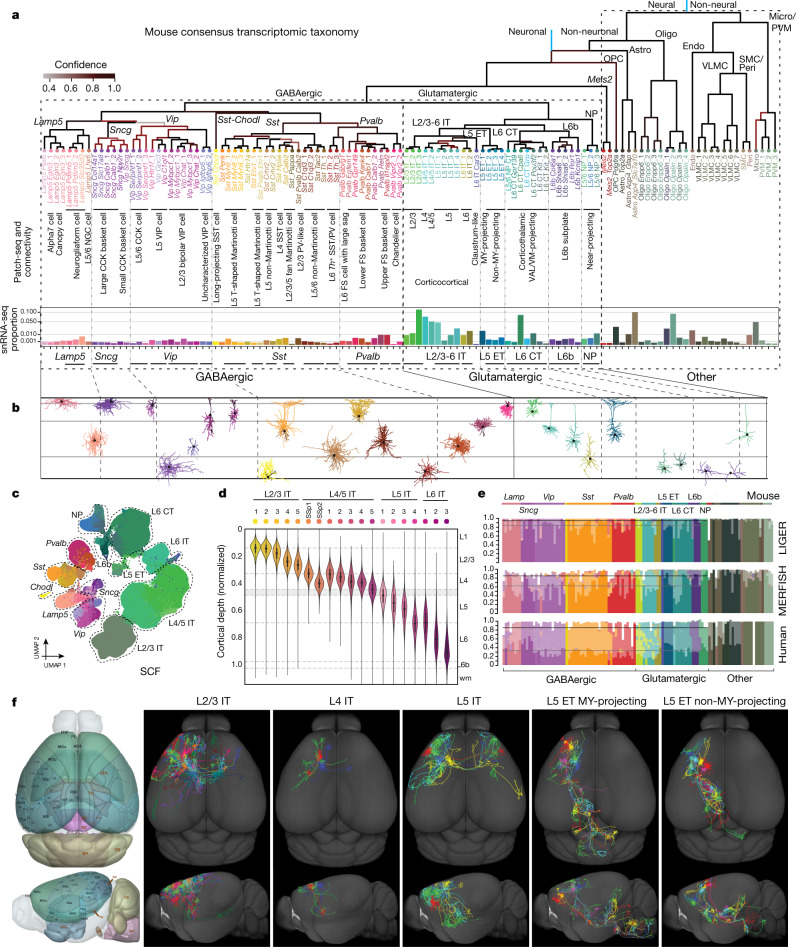
An integrated multimodal census and atlas of MOp cell types. **a**, Mouse MOp consensus transcriptomic taxonomy at the top is used to anchor cell-type features in all the other modalities. Major cellular divisions, class and subclass labels are shown above major branches and cluster labels are shown below each leaf node. Using Patch-seq and connectivity studies, many transcriptomic neuron types or subclasses are annotated and correlated with known cortical neuron types. No Patch-seq data were collected for the ‘uncharacterized’ *Vip* types. Relative proportions of all cell types are calculated from the snRNA-seq 10x v3 B data (bar graph). **b**, Representative local dendritic and axonal morphologies of GABAergic and glutamatergic neuron types from Patch-seq data. **c**, UMAP representation of the mouse transcriptomic–epigenomic integrated molecular taxonomy (SCF version). **d**, Gradual transition of MERFISH IT clusters across cortical layers and depth. **e**, Percentage of LIGER, MERFISH and human cells assigned to mouse consensus transcriptomic cell types (Methods). Darker subclass colours indicate an exact match to the cluster/type, while lighter-coloured stacked bars indicate a match to taxonomic neighbours within the same subclass or, occasionally, a neighbouring subclass. Grey line, mean exact type match over neuronal types; black line, mean subclass match. **f**, Single-neuron full morphology reconstructions show distinct long-range axon projection patterns between glutamatergic subclasses and cell-to-cell variations within each subclass: L2/3 IT (17 cells), L4 IT (3 cells), L5 IT (5 cells), L5 ET MY-projecting (6 cells) and L5 ET non-MY-projecting (6 cells). Left, Allen mouse brain CCFv3 as an anatomical reference. CCK, cholecystokinin; FS, fast spiking; NGC, neurogliaform cell; PV, parvalbumin; SST, somatostatin; VIP, vasoactive intestinal peptide.

As the conclusion of this series of studies from the BICCN, we present an overview and integrated synthesis of the multimodal census and atlas of cell types in the primary motor cortex of mouse, non-human primate and human (Fig. 9).

This integrated synthesis uses the mouse MOp consensus transcriptomic taxonomy^37^ as the anchor (Fig. 9a) because it was derived from the largest datasets and was the reference taxonomy for nearly all the cross-modality and cross-species comparisons. This taxonomy has a hierarchical organization, with major divisions first between neural and non-neural cell types, then between neuronal and non-neuronal types within the neural branch, and finally between GABAergic and glutamatergic types within the neuronal branch.

**Extended Data Fig. 5:**
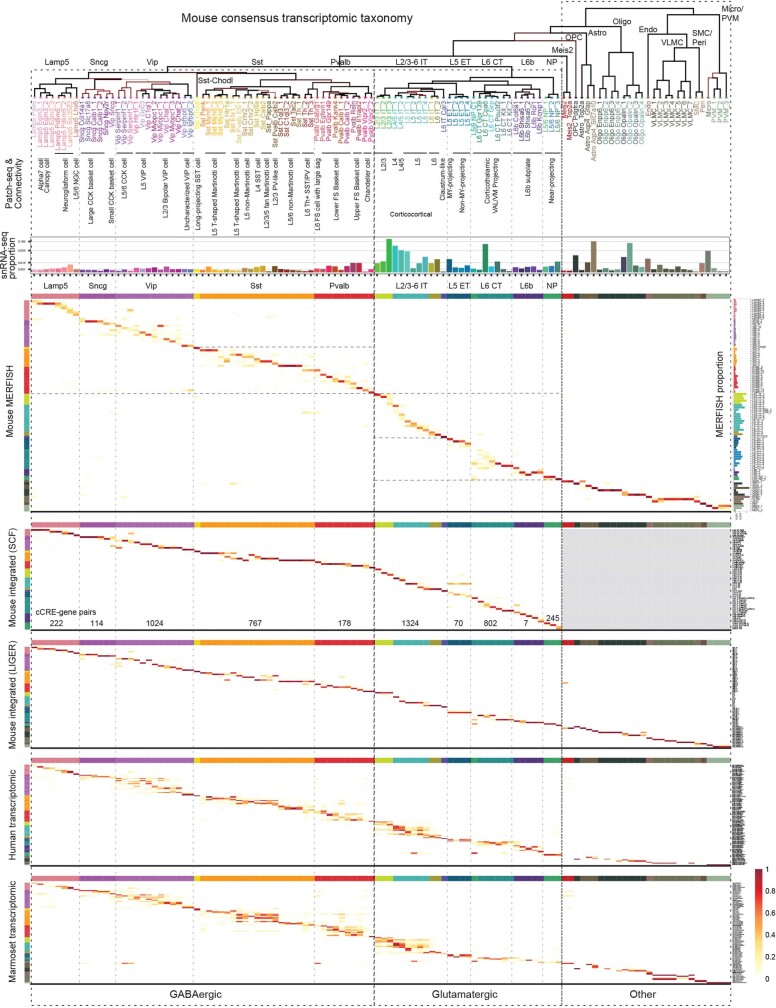
An integrated multimodal census and atlas of cell types in the primary motor cortex of mouse, marmoset and human. The mouse MOp consensus transcriptomic taxonomy at the top is used to anchor cell type features in all the other modalities. Subclass labels are shown above major branches and cluster labels are shown below each leaf node. Confusion matrices show correspondence between the mouse MOp transcriptomic taxonomy (116 clusters) with those derived from other molecular datasets, including mouse MERFISH (95 clusters), the integrated mouse molecular taxonomies by SingleCellFusion (SCF) (56 neuronal clusters) or LIGER (71 clusters), and the human and marmoset transcriptomic taxonomies (127 and 94 clusters, respectively). Cells within each taxonomy were either mapped to the reference (MERFISH, SCF, LIGER) or shared common cells via integration (Human, Marmoset). Color code corresponds to the fraction of cells in each column mapped to or shared with each reference cluster, and each column summed up to 1. These mapping relationships between the mouse consensus transcriptomic taxonomy and other taxonomies are summarized in an overview panel in Figure 9e. Using Patch-seq and connectivity studies, many transcriptomic neuronal types or subclasses are annotated and correlated with known cortical neuron types traditionally defined by electrophysiological, morphological and connectional properties. Relative proportions of all cell types within the mouse MOp are calculated from either the snRNA-seq 10x v3 B dataset (horizontal bar graph) or the MERFISH dataset (vertical bar graph to the right of the MERFISH matrix). The numbers of cCRE-gene pairs in modules corresponding to neuronal subclasses identified by Cicero from the scRNA-seq and snATAC-seq datasets are shown at the bottom of the SCF matrix.

Correspondence matrices show that the mouse MERFISH-based spatial transcriptomic taxonomy^41^, the transcriptomic–epigenomic integrated mouse molecular taxonomies using either SCF or LIGER^37^ and the human and marmoset transcriptomic taxonomies^38^ all aligned largely consistently with the mouse consensus transcriptomic taxonomy (Fig. 9e, Extended Data Fig. 5, Supplementary Table 1). The alignments are highly consistent at subclass level, but disagreements exist at individual-cluster level and increase with cross-species comparison (Fig. 9e), suggesting that differential variations exist in different data types and consistency, in particular that across species, may be more appropriately described at an intermediate level of granularity. We developed a standardized nomenclature system to track cell types described in different modalities (Supplementary Table 2).

Through integrative approaches such as Patch-seq^42^, Epi-retro-seq^45^ and axon projection mapping^44,46^, we related many t-types or subclasses to cortical neuron types traditionally defined by electrophysiological, morphological and connectional properties (Fig. 9a, b, f), thus bridging the cell-type taxonomy with historical knowledge. We derived the relative proportion of each cell type in mouse MOp using either snRNA-seq or MERFISH data. The MERFISH data^41^ also revealed the spatial distribution pattern of each cell type, showing that many glutamatergic or GABAergic neuron types adopt narrow distributions along the cortical-depth direction, often occupying predominantly a single layer or a sublayer, and related types (for example, the L2/3-6 IT excitatory types) display a largely gradual transition across cortical depth or layers (Fig. 9d).

**Extended Data Fig. 6:**
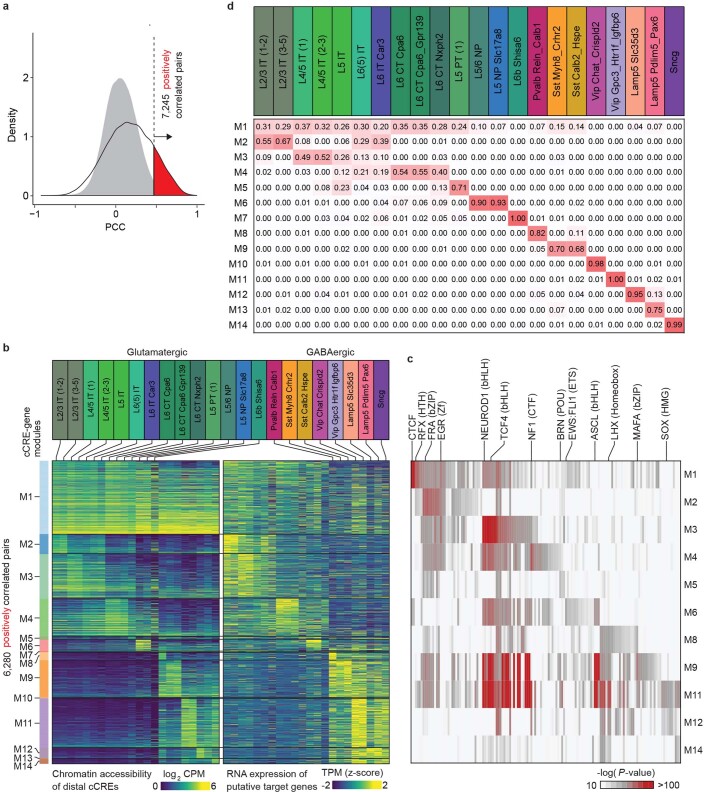
Identification of putative enhancer-gene pairs. **a**, Detection of putative enhancer-gene pairs. 7,245 pairs of positively correlated cCRE and genes (highlighted in red) were identified using an empirically defined significance threshold of FDR < 0.01. Grey filled curve shows the distribution of PCC for randomly shuffled cCRE-gene pairs. **b**, Heatmap of chromatin accessibility of 6,280 putative enhancers, grouped by distinct enhancer-gene modules, across joint cell clusters (left) and expression of 2,490 target genes (right). Note genes are displayed for each putative enhancer separately. CPM: counts per million, TPM: transcripts per million. About 76% of putative enhancers showed cluster-specific chromatin accessibility and were enriched for lineage-specific TFs, while 24% were widely accessible and linked to genes expressed across neuronal clusters with the highest expression in glutamatergic neurons (module M1). Other modules (M2 to M14) of enhancer-gene pairs were active in a subclass-specific manner. **c**, Enrichment of known TF motifs in distinct enhancer-gene modules. Displayed are known motifs from HOMER with enrichment -log p-value > 10. In module M1, de novo motif analysis of putative enhancers showed enrichment of sequence motifs recognized by TFs CTCF and MEF2. CTCF is a widely expressed DNA binding protein with a well-established role in transcriptional insulation and chromatin organization, but recently it was also reported that CTCF can promote neurogenesis by binding to promoters and enhancers of related genes. In the L2/3 IT selective module M2, putative enhancers were enriched for the binding motif for Zinc-finger transcription factor EGR, a known master transcriptional regulator of excitatory neurons^89^. In the Pvalb selective module M8, putative enhancers were enriched for sequence motifs recognized by the MADS factor MEF2, which is associated with regulating cortical inhibitory and excitatory synapses and behaviours relevant to neurodevelopmental disorders^90^. **d**, Heatmap showing the weight of each joint cell cluster in each module, derived from the coefficient matrix. The values of each column are scaled (0–1).

**Extended Data Fig. 7:**
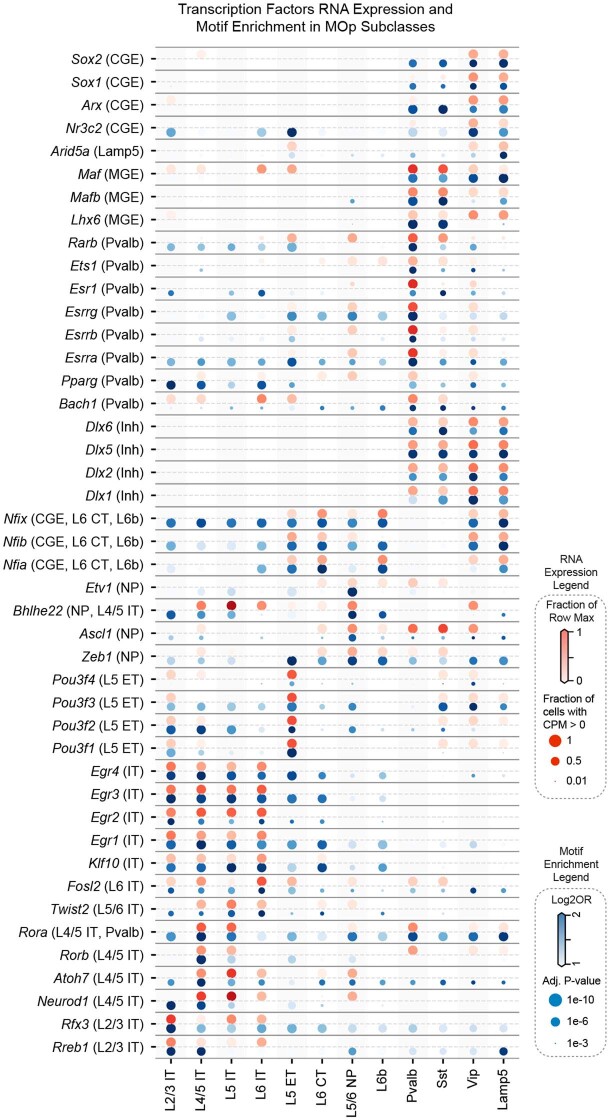
Dot plot illustrating RNA expression levels (red) and hypo-CG-DMR motif enrichments (blue) of transcription factors (TFs) in mouse MOp subclasses. The size and color of red dots indicate the proportion of expressing cells and the average expression level in each subclass, respectively. The size and color of blue dots indicate adjusted P-value (Fisher’s exact test, Benjamini-Hochberg Procedure) and log_2_(Odds Ratio) of motif enrichment analysis, respectively. Combining these two orthologous pieces of evidence identified many well-studied TFs in embryonic precursors, such as the *Dlx* family members for pan-inhibitory neurons, and *Lhx6* and *Mafb* for MGE derived inhibitory neurons. We further identified many additional TFs with more restricted patterns in specific subclasses, such as *Rfx3* and *Rreb1* (in L2/3 IT), *Atoh7* and *Rorb* (in L4/5 IT), *Pou3* family members (in L5 ET), *Etv1* (in L5/6 NP), *Esrr* family members (in *Pvalb*), and *Arid5a* (in *Lamp5*).

Finally, we demonstrate the potential to elucidate gene regulatory mechanisms by discovering candidate CREs (cCREs) and master transcription factors specific to neuronal subclasses in the combined transcriptomic and epigenomic datasets (Fig. 9c). We found 7,245 distal (more than 1 kbp from the transcription start site) cCRE–gene pairs in MOp neurons that showed a positive correlation between accessibility at 6,280 cCREs and expression level of 2,490 putative target genes (Methods)^37,40^. We grouped these putative enhancers into modules based on accessibility across cell clusters (Extended Data Fig. 6) and identified a large number of enhancer–gene pairs for each subclass of neurons (Extended Data Fig. 5). Similarly, we identified transcription factors showing cell-type specificity supported by both RNA expression and DNA-binding motif enrichment in cell subclasses^37,39^ (Methods) (Extended Data Fig. 7).

## Discussion

### A cell census and atlas of primary motor cortex

Understanding the principles of brain circuit organization requires a detailed understanding of its basic components. The current effort combines a wide array of single-cell-based techniques to derive a robust and comprehensive molecular cell-type classification and census of the primary motor cortex of mouse, marmoset and human, coupled with a spatial atlas of cell types and an anatomical input–output wiring diagram in mouse. We demonstrate the robustness and validity of this classification through strong correlations across cellular phenotypes, and strong conservation across species. Together these data comprise a cell atlas of the primary motor cortex that encompasses a comprehensive reference catalogue of cell types, their proportions, spatial distributions, anatomical and physiological characteristics, and molecular genetic profiles, registered into a CCF. This cell atlas establishes a foundation for an integrative study of the architecture and function of cortical circuits akin to reference genomes for studying gene function and genome regulatory architecture. Furthermore, it provides a map of the genes that contribute to cellular phenotypes and their epigenetic regulation. These data resources and associated tools enabling genetic access for manipulative experimentation are publicly available. This body of work provides a roadmap for exploring cellular diversity and organization across brain regions, organ systems and species.

### Principles of cortical cell-type organization

Substantiating previous studies^9,10^, our multimodal cross-species study of the primary motor cortex suggests that a general principle of cortical cell-type organization is its hierarchical relationship, whereby high-level classes linked by major branches comprise progressively finer subpopulations connected by minor branches. In this scheme, the higher-level classes and subclasses are categorically and concordantly distinct from each other across modalities, are conserved across species, and probably arise from different developmental programs, such as GABAergic neuron derivatives of different zones of the ganglionic eminences or the layer-selective glutamatergic neurons derived sequentially from progenitors of the cortical plate. At the lower branch levels (types or clusters), however, while certain cell types are highly distinct (for example, *Pvalb* chandelier cells), distinctions and boundaries among many other clusters can be ambiguous and vary among different modalities.

In this context, another important finding, consistent with and building on multiple other studies^9,11,68,69^, is the coexistence of discrete and continuous variations of cell features across modalities at the lower branch level. A compelling example is the continuous and concordant variation of transcriptomic, anatomical and physiological properties along cortical depth within multiple cell populations, including the glutamatergic L2/3–L6 IT and GABAergic *Sst* and *Pvalb* subclasses. Although some of the variations may result from technical factors, such as differences in the resolution of measurements across data modalities (with transcriptomics providing the highest granularity at present), a major source of these continuous variations may reflect true biology, supported by the coordinated variation across transcriptomic, spatial, morphological and physiological properties as shown by MERFISH and Patch-seq. Therefore, another emerging principle of cell type organization is the coexistence of discrete and continuous variations that underlie cell-type diversity.

Together, the principles of hierarchical organization comprising discrete classes and types as well as continuum within and across subpopulations represent a more nuanced and biologically realistic description of cell-type landscape, with implications in cell classification and census. For example, the multimodal variations at finer granularity may preclude a fully discretized representation of cell types with consistency across cell phenotypes, and may explain some of the discrepancies in estimated numbers of cell types using different approaches. An intriguing question is whether continuous variations of cell features will increase further or become more discretized in the context of neural circuit operation, converging to a set of distinct functional elements from a more continuous cellular landscape. An example of this is regionalization. We identify a MOp-specific input–output wiring diagram—however, transcriptomic cell types are generally shared between MOp and its neighbouring cortical areas^11^. Region-specific connectivity patterns of similar molecular types may be a major factor defining the functional specificity of the primary motor cortex.

### Perspectives on cell-type classification

Our findings have major implications for understanding the biological basis of cellular identity towards a more rigorous, quantitative and satisfying definition and classification of cell types. First and foremost, our discovery of the compelling correspondence across molecular genetic, anatomical and physiological features of hierarchically organized cell populations, reflecting developmental origins and mainly conserved across mammalian species, demonstrates the biological validity and genomic underpinning of major cell types. These findings establish a unifying and mechanistic framework of cell-type classification that integrates multi-layered molecular genetic information with multi-faceted phenotypic properties. Thus, single-cell transcriptomics and epigenomics can serve as powerful approaches for establishing a foundational framework of cell types, owing to not only their unparalleled scalability but also to their representation of the underlying molecular genetic programs rooted in development and evolution. Physiological, morphological and connectional characterizations assign functional attributes to cells; their concordance with molecular identities provides strong validation to the molecularly defined cell types, whereas their differential variations reveal additional, probably network- and activity-driven factors that contribute to further refinement of cell types.

While the higher levels of the hierarchy comprise ~around 25 subclasses (16 neuronal and 9 non-neuronal) that are identified with remarkable consistency across multiple species and experimental modalities, many finer levels of cell properties do not neatly segregate into discrete and consistent sets of cell types with perfect correspondence among data modalities. These include aspects of continuous distributions, species specializations and mismatches between molecular and anatomical phenotypes that may result from developmental events no longer represented in the adult. Different methods provide somewhat different granularity of clustering, and thus different numbers of putative cell types. For example, single-cell transcriptomics identifies around 100 clusters representing the terminal leaves of this hierarchically branched organization^37^. Looking ahead, it is important to note that at more refined levels, the number of cell types that can be distinguished will probably change with additional cellular features characterized at greater breadth and depth using new methods and approaches.

Overall, the landscape of cell types appears to be generated from a combination of specification through evolutionarily driven and developmentally regulated genetic mechanisms, and refinement of cellular identities through intercellular interactions within the network in which the cells are embedded. In this scenario, genetic mechanisms drive intrinsic or cell-autonomous determination of cell fate, as well as progressive temporal generation of cell types from common progenitor pools that explain global similarities and continuous features of cellular phenotypes reflecting developmental gradients. Network influences can drive further phenotypic refinement that may not be reflected in the adult genetic signature—for example for axonal projection and synaptic connectivity that may reflect transient or stochastic developmental events, region or circuit-specific and/or activity or plasticity-dependent modification to form and reshape functionally specific circuits. Future studies focusing on these mechanisms and testing of the ensuing hypotheses will enable a deeper understanding of the nature of variability among related cell types in the mammalian brain.

### Cell-type conservation and divergence

Evolutionary conservation is strong evidence of functional significance. The demonstrated conservation of cell types from mouse, marmoset, macaque and human suggests that these conserved types have important roles in cortical circuitry and function in mammals and even more distantly related species. We also find that similarity of cell types varies as a function of evolutionary distance, with substantial species differences that represent either adaptive specialization or genetic drift. For the most part, species specializations tend to appear at the finer branches of the hierarchical taxonomy. This result is consistent with a recent hypothesis in which cell types are defined by common evolutionary descent and evolve independently, such that new cell types are generally derived from existing genetic programs and appear as specializations at the finer levels of the taxonomic tree^70^.

A surprising finding across all homologous cell types was the relatively high degree of divergence for genes with cell-type-specific expression in a given species. This observation provides a clear path to identify core conserved genes underlying the canonical identity and features of those cell types. Furthermore, it highlights the need to understand species adaptations superimposed on the conserved program, as many specific cellular phenotypes may vary across species including gene expression, epigenetic regulation, morphology, connectivity and physiological properties. As we illustrated in the Betz cells, there is clear homology across species in the L5 ET subclass, but variation in many measurable properties across species.

### Linking model organisms to human biology and disease

Our findings have major implications for the consideration of model organisms to understand human brain function and disease. Despite major investments, animal models of neuropsychiatric disorders have often been characterized by ‘loss of translation’, fuelling heated debates about the utility of model organisms in the development of treatments for human diseases. Cell census information aligned across species will be highly valuable for making rational choices about the best models for each disease and therapeutic target. For example, the characterization of cell types and their properties shown in Fig. 9 can be used to infer the main characteristics of homologous cell types in humans and other mammalian species, which would be difficult to obtain otherwise. They can also reveal potential limitations of model organisms and the necessity to study human and other primates to understand the specific cell-type features that contribute to human brain function and diseases. This reductionist dissection of the cellular components provides a foundation for understanding the general principles of neural circuit organization and computation that underlie mental activities and brain disorders.

### Future directions

The approach we took to generate a cell census and atlas through a systematic dissection of cell types opens up numerous avenues for future work. The MOp census and atlas provides a foundational platform for the broad neuroscience community to accumulate and integrate cell-type information across species. Classification of cell types based on their molecular, spatial and connectional properties in the adult sets the stage for developmental studies to understand the molecular genetic programs underlying cell-type specification, maturation and circuit assembly. The molecular genetic information promises to deliver tools for genetic access to many brain cell types via transgenic and enhancer virus strategies. A combination of single-cell transcriptomics and functional measurements may further elucidate the roles of distinct cell types in circuit computation during behaviour, bridging the gap between molecular and functional definition of cell types. The systematic, multi-modal strategy described here can be extended to the whole brain, and major efforts are underway in the BICCN to generate a brain-wide cell census and atlas in the mouse with increasing coverage of human and non-human primates.

## Methods

### Nomenclature of the L5 ET subclass of glutamatergic neurons

In this manuscript we have adopted a nomenclature for major subclasses of cortical glutamatergic excitatory neurons, which have long-range projections both within and outside of the cortex, following a long tradition of naming conventions that often classify neurons based on their projection targets. This nomenclature is based on our de novo transcriptomic taxonomy (Fig. 9) that organizes cell types hierarchically and validates the naming of the primary branches of glutamatergic neurons by their major long-range projection targets. At these levels, glutamatergic neurons are clearly divided into several subclasses, the cortico-cortical and cortico-striatal only projecting IT neurons that are distributed across nearly all layers (L2/3 IT, L4/5 IT, L5 IT, L6 IT and L6 IT *Car3*), the layer 5 neurons projecting to extratelencephalic targets (L5 ET), the CT-projecting neurons in layer 6 (L6 CT), the NP neurons found in layers 5 and 6, and the L6b neurons whose projection patterns remain largely unknown.

While the IT, CT, NP and L6b neurons have been consistently labelled as such in the field, the L5 ET neurons have not been named consistently in the literature, largely owing to their large variety of projection targets and other phenotypic features that vary depending on cortical areas and species. Here we use the term L5 ET (layer 5 extratelencephalic) to refer to this prominent and distinct subclass of neurons as a standard name that can be accurately used across cortical regions and across species, and we provide our rationale below.

It has long been appreciated that cortical layer 5 contains two distinct populations of neurons that can be distinguished, not only based on the presence or absence of projections to ET targets (ET and IT cells), but also based on their predominant soma locations, dendritic morphologies and intrinsic physiology^48^. Accordingly, various names incorporating these features have been adopted to refer to L5 ET versus L5 IT cells, such as L5b versus L5a, thick-tufted versus thin-tufted and burst-firing versus regular-firing. The most common term used to refer to L5 ET cells residing in motor cortical areas has been PT, which refers to neurons projecting to the pyramidal tract. As accurately stated in Wikipedia, “The pyramidal tracts include both the corticobulbar tract and the corticospinal tract. These are aggregations of efferent nerve fibers from the upper motor neurons that travel from the cerebral cortex and terminate either in the brainstem (corticobulbar) or spinal cord (corticospinal) and are involved in the control of motor functions of the body.”

Owing to the past wide use of the term PT, we do not take the decision to use L5 ET rather than PT lightly. However, in the face of multiple lines of evidence that have accumulated over the last several years^72,73^ and prominently highlighted in this manuscript, it is now clear that PT represents only a subset of L5 ET cells and is thus unable to accurately encompass the entire L5 ET subclass. This realization is informed by comparisons across species and cortical areas, and by single-cell transcriptomics and descriptions of the projections of single neurons, as well as studies linking transcriptional clusters to projection targets.

As noted above, the overall transcriptomic relationships between cortical neurons are well-described by a hierarchical tree that closely matches developmental lineage relationships as neurons become progressively restricted in their adult fates^37,38^ (Fig. 9). The cortical excitatory neurons are a major branch, distinct from inhibitory, glial and epithelial cells. Subsequent splitting of the excitatory neurons reveals several major excitatory neuron subclasses—IT, L5 ET, L6 CT, NP and L6b. These major subclasses are conserved across mammalian species^9,10^, as well as across all cortical areas as shown in mouse^11^. It is therefore clear that names are needed that both accurately incorporate and accurately distinguish between neurons in these subclasses, and which are applicable across all cortical areas.

Also as noted above, a widely used alternative to L5 ET is PT. Further, this term is traditionally used along with CT to distinguish between cells with these different projections. The two main observations that make these alternative nomenclatures untenable are: (1) PT refers to motor neurons that project into MY or spinal cord, but in many cortical areas (for example, visual and auditory areas) none of the L5 ET cells are motor neurons; and (2) even in the motor cortex many cells in the L5 ET subclass do not project to the pyramidal tract and instead project solely to the TH (or to TH and other non-PT targets). This is revealed by single-neuron reconstructions^18,46,53^ (Figs. 6, 8), BARseq^64^, projections from neuron populations with known gene expression and anatomical position in mouse lines^63^, and studies directly linking projections to transcriptomics^9,41^ and epigenetics^45^ (Figs. 4, 8). The term PT therefore is not inclusive of the entire L5 ET subclass. Furthermore, the L5 CT cells within the L5 ET subclass are largely continuous with PT cells (or ‘PT-like’ cells), not only genetically but also anatomically^41,42^ (Figs. 2, 3), as a majority of L5 ET cells project to multiple targets, typically including both the TH and the PT structures (for example, MY and spinal cord), as well as the midbrain^46^ (Figs. 6, 8). Thus, the L5 ET subclass should neither be split into PT and CT, nor should the CT-only cells be omitted by use of the term PT. These facts also inform us that it is important to maintain a distinction between L5 CT (a type of L5 ET) and L6 CT (a major subclass of cortical excitatory neurons that is highly distinct from L5 ET, despite the presence of some L6 CT cells at the bottom of layer 5)^41^. CT can be accurately used as a generic term, but CT neurons do not belong to a single subclass of cortical excitatory neurons.

We recognize that another name that has been used to describe L5 ET cells is subcerebral projection neuron (SCPN)^49^. Given that the telencephalon is equivalent to the cerebrum, ET and subcerebral have the same meaning and the term L5-SCPN would be an accurate and equivalent alternative. But the ‘L5’ qualifier is crucial in either case to distinguish these cells from the L6 CT subclass. We favour the use of ET because SCPN has not been widely adopted and due to symmetry with the widely used ‘IT’ nomenclature. Alternatively, given their evidence that “unlike pyramidal tract neurons in the motor cortex, these neurons in the auditory cortex do not project to the spinal cord”, Chen et al^64^ used the term ‘pyramidal tract-like’ (PT- l). We also favour L5 ET over L5 PT-l which clings to an inaccurate and now outdated nomenclature.

### Integrating 10x v3 snRNA-seq datasets across species

To identify homologous cell types across species, human, marmoset and mouse 10x v3 snRNA-seq datasets were integrated using Seurat’s SCTransform workflow. Each major cell class (glutamatergic, GABAergic and non-neuronal cells) was integrated separately across species. Expression matrices were reduced to 14,870 one-to-one orthologues across the three species (NCBI Homologene; 22 November 2019). Nuclei were downsampled to have approximately equivalent numbers at the subclass level across species. Marker genes were identified for each species cluster using Seurat’s FindAllMarkers function with test.use set to ‘roc’, > 0.7 classification power. Markers were used as input to guide alignment and anchor-finding during integration steps. For full methods see ref. ^38^. Code for generating Figs. 1b–h, 3, Extended Data Fig. 2 is available at http://data.nemoarchive.org/publication_release/Lein_2020_M1_study_analysis/Transcriptomics/flagship/. Analysis was performed in RStudio using R version 3.5.3, R packages: Seurat 3.1.1, ggplot2 3.2.1 and scrattch.hicat 0.0.22.

### Estimation of cell-type homology

To establish a robust cross-species cell type taxonomy, we applied a tree-based clustering method on integrated class-level datasets (https://github.com/AllenInstitute/BICCN_M1_Evo). The integrated space (from the previously mentioned Seurat integration) was over-clustering into small sets of highly similar nuclei for each class (about 500 clusters per class). Clusters were aggregated into metacells, then hierarchical clustering was performed based on the metacell gene expression matrix using Ward’s method. Hierarchical trees were then assessed for cluster size, species mixing and branch stability by subsampling the dataset 100 times with 95% of nuclei. Finally, we recursively searched every node of the tree, and if certain heuristic criteria were not sufficient for a node below the upper node, all nodes below the upper node were pruned and nuclei belonging to this subtree were merged into one homologous group. We identified 24 GABAergic, 13 glutamatergic and 8 non-neuronal cross-species consensus clusters that were highly mixed across species and robust. For full methods see ref. ^38^. A final dendrogram of consensus cell types was constructed by transforming the raw unique molecular identifier (UMI) counts to log_2_(counts per million (CPM)) normalized counts. Up to 50 marker genes per cross-species cluster were identified by using the scrattch.hicat (v0.0.22) (https://github.com/AllenInstitute/scrattch.hicat) display_cl and select_markers functions with the following parameters; q1.th = 0.4, q.diff.th = 0.5, de.score.th = 80. Median cross-species cluster log_2_ CPM expression of these genes were then used as input for scrattch.hicat’s build_dend function. This analysis was bootstrapped 10,000 times with branch colour denoting confidence. Branch robustness was assessed by rebuilding the dendrogram 10,000 times with a random 80% subset of variable genes across clusters and calculating the proportion of iterations that clusters were present on the same branch. Consensus taxonomy agreement in Fig. 9e is determined by selecting maximum frequency leaf match with stacked bars indicating assigned consensus cell types in the centred neighbourhood.

### Cross-species differential gene expression and correlations

Expression matrices were subsetted to include one-to-one orthologous genes across all three species. Spearman correlations shown in Fig. 1d were performed by comparing cross-species cluster median log_2_ CPM expression of all orthologous genes for each species pair. To calculate the number of differentially expressed genes between each species pair for each cross-species cluster, we used a pseudobulk comparison method^74^ from DESeq2 (v1.30.0). For a given cross-species cluster, each sample was split by species and donor, then a Wald test was performed between each species pair. Genes with adjusted *P*-values < 0.05 and log_2_ fold-changes greater than 2 in either direction were counted and reported in Fig. 1e.

### Generation of Epi-retro-seq data

We injected retrograde tracer rAAV2-retro-Cre^75^ into a target region in INTACT mice^76^, which turned on Cre-dependent GFP expression in the nuclei of MOp neurons projecting to the injected target region. Individual GFP-labelled nuclei of MOp projection neurons were then isolated using fluorescence-activated nucleus sorting (FANS) (box outlines selected cells in Fig. 4a). snmC-seq2^77^ was performed to profile the DNA methylation (mC) of each single nucleus.

### Evaluation of contamination in Epi-retro-seq

The methods used to evaluate contamination level and potential reasons are described in detail in ref. ^45^. Specifically, we quantified the ratio between the number of cells in expected on-target subclasses (for example, L5 ET cluster for ET-projecting neurons) versus in expected off-target subclasses (for example, IT clusters for ET-projecting neurons), denoted as *r*_*p*_, and compared the ratio with the one expected from the unbiased data without enrichment for specific projections, denoted as *r*_*u*_. This provides an estimation of signal-to-noise ratio of each FANS experiment. For IT projections, we used IT subclasses as on-target and L6 CT + inhibitory as off-target, and for ET projections, we used L5 ET as on-target and IT + inhibitory as off-target. For the MOp neurons without enrichment of projections, the expected ratio between cells in IT subclasses and in L6 CT + inhibitory are *r*_*u *_= 2,652:1,775, whereas the expected ratio between cells in L5 ET subclass and in IT + inhibitory are *r*_*u *_= 202:3,434. The fold enrichment in the text was computed by *r*_*p*_/*r*_*u*_ for each FANS run separately and averaged across IT or ET targets respectively.

We want to point out that, in addition to this computational method, other methods are available to evaluate and minimize potential contamination in Epi-retro-seq. In cases in which differences in expected results from on- versus off-target populations are unknown, other available methods would need to be used to eliminate cases in which injections might have directly labelled cells outside the intended target region, such as examination of labelling along the injection electrode track.

### Integration of L5 ET cells from Epi-retro-seq and 10x snRNA-seq

For snRNA-seq, the 4,515 cells from 10x v3 B dataset labelled as L5 ET by SCF were selected^37^. The read counts were normalized by the total read counts per cell and log transformed. Top 5,000 highly variable genes were identified with Scanpy^78^ (v1.8.1) and *z*-score was scaled across all the cells. For Epi-retro-seq, the posterior methylation levels of 12,261 genes in the 848 L5 ET cells were computed^45^. Top 5,000 highly variable genes were identified with AllCools^79^ and *z*-score was scaled across all the cells. The 1,512 genes as the intersection between the two highly variable gene lists were used in Scanorama^80^ (v1.7.1) to integrate the *z*-scored expression matrix and minus *z*-scored methylation matrix with sigma equal to 100.

### Integrating mouse transcriptomic, spatially resolved transcriptomic, and epigenomic datasets

To integrate IT cell types from different mouse datasets, we first take all cells that are labelled as IT, except for L6_IT_Car3, from the 11 datasets as listed in Fig. 7a. These cell labels are either from dataset-specific analyses^41,45^, or from the integrated clustering of multiple datasets^37^. The integrated clustering and embedding of the 11 datasets are then generated by projecting all datasets into the 10x v2 scRNA-seq dataset using SingleCellFusion^37,79^. Genome browser views of IT and ET cell types (Figs. 7b, 8c) are taken from the corresponding cell types of the brainome portal^37^ (https://brainome.ucsd.edu/BICCN_MOp). MERFISH data were analysed using custom Python code, which is available at https://github.com/ZhuangLab/MERlin.

### Identification of cCREs

For peak calling in the snATAC-seq data, we extracted all the fragments for each cluster, and then performed peak calling on each aggregate profile using MACS2^81^ v2.2.7.1. using Python 3.6 with parameter: “--nomodel --shift −100 --ext 200 --qval 1e-2 –B --SPMR”. First, we extended peak summits by 250 bp on either side to a final width of 501 bp. Then, to account for differences in performance of MACS2 based on read depth and/or number of nuclei in individual clusters, we converted MACS2 peak scores (−log_10_(*q*-value)) to ‘score per million’^82^. Next, a union peak set was obtained by applying an iterative overlap peak-merging procedure, which avoids daisy-chaining and still allows for use of fixed-width peaks. Finally, we filtered peaks by choosing a score per million cut-off of 5 as cCREs for downstream analysis.

### Predicting enhancer–promoter interactions

First, co-accessible cCREs are identified for all open regions in all neuron types (cell clusters with less than 100 nuclei from snATAC-seq are excluded) using Cicero^83^ with the following parameters: aggregation k = 50, window size = 500 kb, distance constraint = 250 kb. In order to find an optimal co-accessibility threshold, we generated a random shuffled cCRE-by-cell matrix as background and calculated co-accessible scores from this shuffled matrix. We fitted the distribution of co-accessibility scores from random shuffled background into a normal distribution model by using the R package fitdistrplus^84^. Next, we tested every co-accessible cCRE pair and set the cut-off at co-accessibility score with an empirically defined significance threshold of FDR < 0.01. The cCREs outside of ±1 kb of transcriptional start sites in GENCODE mm10 (v16) were considered distal. Next, we assigned co-accessibility pairs to three groups: proximal-to-proximal, distal-to-distal and distal-to-proximal. In this study, we focus only on distal-to-proximal pairs. We calculated the Pearson’s correlation coefficient (PCC) between gene expression (scRNA SMART-seq) and cCRE accessibility across the joint clusters to examine the relationships between the distal cCREs and target genes as predicted by the co-accessibility pairs. To do so, we first aggregated all nuclei or cells from scRNA-seq and snATAC-seq for every joint cluster to calculate accessibility scores (log_2_ CPM) and relative expression levels (log_2_ transcripts per million). Then, PCC was calculated for every gene-cCRE pair within a 1-Mbp window centred on the transcriptional start sites for every gene. We also generated a set of background pairs by randomly selecting regions from different chromosomes and shuffling the cluster labels. Finally, we fit a normal distribution model on background and defined a cut-off at PCC score with an empirically defined significance threshold of FDR < 0.01, in order to select significant positively correlated cCRE-gene pairs.

### Identification of *cis*-regulatory modules

We used nonnegative matrix factorization (NMF) to group cCREs into *cis*-regulatory modules based on their relative accessibility across cell clusters. We adapted NMF (Python package: sklearn v.0.24.2) to decompose the cluster-by-cCRE matrix *V* (*N* × *M*, *N* rows: cCRE, *M* columns: cell clusters) into a coefficient matrix *H* (*R* × *M*, *R* rows: number of modules) and a basis matrix *W* (*N* × *R*), with a given rank *R*: *V* ≈ *WH*.

The basis matrix defines module related accessible cCREs, and the coefficient matrix defines the cell cluster components and their weights in each module. The key issue to decompose the occupancy profile matrix was to find a reasonable value for the rank *R* (that is, the number of modules). Several criteria have been proposed to decide whether a given rank *R* decomposes the occupancy profile matrix into meaningful clusters. Here we applied a measurement called sparseness^85^ to evaluate the clustering result. Median values were calculated from 100 times for NMF runs at each given rank with a random seed, which will ensure the measurements are stable. Next, we used the coefficient matrix to associate modules with distinct cell clusters. In the coefficient matrix, each row represents a module and each column represents a cell cluster. The values in the matrix indicate the weights of clusters in their corresponding module. The coefficient matrix was then scaled by column (cluster) from 0 to 1. Subsequently, we used a coefficient > 0.1 (~95th percentile of the whole matrix) as a threshold to associate a cluster with a module. Similarly, we associated each module with accessible elements using the basis matrix. For each element and each module, we derived a basis coefficient score, which represents the accessible signal contributed by all clusters in the defined module.

### Identification of subclass-selective transcription factors by both RNA expression and motif enrichment

All analyses for this section were at the subclass level. For RNA expression, we used the scSMART-seq dataset and compared each subclass with the rest of the population through a one-tailed Wilcoxon test and FDR correction to select significantly differentially expressed transcription factors (adjusted *P*-value < 0.05, cluster average fold change > 2). To perform the motif enrichment analysis, we used known motifs from the JASPAR 2020 database^86^ and the subclass specific hypo-CG-DMR identified in Yao et al.^37^. The AME software from the MEME suite (v5.1.1)^87^ was used to identify significant motif enrichment (adjusted *P*-value < 10^−3^, odds ratio > 1.3) using default parameters and the same background region set as described^37^. All genes in Extended Data Fig. 7 were both significantly expressed and had their motif enriched in at least one of the subclasses.

### Generation and use of new knockin mouse lines

All experimental procedures were approved by the Institutional Animal Care and Use Committees (IACUC) of Cold Spring Harbor Laboratory, University of California Berkeley and Allen Institute, in accordance with NIH guidelines. Mouse knockin driver lines are being deposited to the Jackson Laboratory for wide distribution.

### Generation and use of *Tle4-2A-CreER*, *Fezf2-2A-CreER*, *PlexinD1-2A-CreER*, *PlexinD1-2A-Flp*, *Tbr2-2A-CreER* and dual-tTA mouse lines

Driver and reporter mouse lines were generated using a PCR-based cloning. Knockin mouse lines *Tle4-2A-CreER*, *Fezf2-2A-CreER*, *PlexinD1-2A-CreER*, *PlexinD1-2A-Flp* and *Tbr2-2A-CreER* were generated by inserting a 2A-CreER or 2A-Flp cassette in-frame before the STOP codon of the targeted gene. Targeting vectors were generated using a PCR-based cloning approach^27,47^. In brief, for each gene of interest, two partially overlapping BAC clones from the RPCI-23&24 library (made from C57BL/b mice) were chosen from the Mouse Genome Browser. 5′ and 3′ homology arms were PCR amplified (2–5 kb upstream and downstream, respectively) using the BAC DNA as template and cloned into a building vector to flank the 2A-CreERT2 or 2A-Flp expressing cassette as described^27^. These targeting vectors were purified, tested for integrity by enzyme restriction and PCR sequencing. Linearized targeting vectors were electroporated into a 129SVj/B6 hybrid ES cell line (v.6.5). ES cell clones were first screened by PCR and then confirmed by Southern blotting using appropriate probes. DIG-labelled Southern probes were generated by PCR, subcloned and tested on wild-type genomic DNA to verify that they give clear and expected results. Positive v6.5 ES cell clones were used for tetraploid complementation to obtain male heterozygous mice following standard procedures. The F_0_ males and subsequent generations were bred with reporter lines (*Ai14*, Snap25-LSL-EGFP, *Ai65*) and induced with tamoxifen at the appropriate ages to characterize the resulting genetically targeted recombination patterns. Drivers *Tle4-2A-CreER*, *Fezf2-2A-CreER* and *PlexinD1-2A-CreER* were additionally crossed with reporter Rosa26-CAG-LSL-Flp and *Tbr2-2A-CreER;PlexinD1-2A-Flp* with reporter dual-tTA, and induced with tamoxifen at the appropriate age to perform anterograde viral tracing, with Flp- or tTA-dependent AAV vector expressing EGFP (AAV8-CAG-fDIO-TVA-EGFP or AAV-TRE-3g-TVA-EGFP), to characterize the resulting axon projection patterns.

### Generation of *Npnt-P2A-FlpO* and *Slco2a1-P2A-Cre* mouse lines

To generate lines bearing in-frame genomic insertions of P2A-FlpO or P2A-Cre, we engineered double-strand breaks at the stop codons of *Npnt* and *Slco2a1*, respectively, using ribonucleoprotein (RNP) complexes composed of SpCas9-NLS protein and in vitro transcribed sgRNA (*Npnt*: GATGATGTGAGCTTGAAAAG and *Slco2a1*: CAGTCTGCAGGAGAATGCCT). These RNP complexes were nucleofected into 10^6^ v6.5 mouse embryonic stem cells (C57/BL6;129/sv; a gift from R. Jaenisch) along with repair constructs in which P2A-FlpO or P2A-Cre was flanked with the following sequences homologous to the target site, thereby enabling homology-directed repair.

*Npnt-P2A-FlpO*: TGGCCCTTGAGCTCTAGTGTTCCCACTTGCCATAGAAATCTGATCTTCGGTTTGGGGGAAGGGTTGCCTTACCATGCTCCATGAGTGAGCACTGGGAAAAGGGGCAGAGGAGGCCTGACCAGTGTATACGTTCTCTCCCTAGGTCATCTTCAAAGGTGAAAAAAGGCGTGGTCACACGGGGGAGATTGGATTGGATGATGTGAGCTTGAAGCGCGGAAGATGTGGAAGCGGAGCTACTAACTTCAGCCTGCTGAAGCAGGCTGGAGACGTGGAGGAGAACCCTGGACCTATGGCTCCTAAGAAGAAGAGGAAGGTGATGAGCCAGTTCGACATCCTGTGCAAGACCCCGCCGAAGGTGCTGGTGCGGCAGTTCGTGGAGAGATTCGAGAGGCCCAGCGGCGAAAAGATCGCCAGCTGTGCCGCCGAGCTGACCTACCTGTGCTGGATGATCACCCACAACGGCACCGCGATCAAGAGGGCCACCTTCATGAGTTATAACACCATCATCAGCAACAGCCTGAGTTTTGACATCGTGAACAAGAGCCTGCAGTTCAAGTACAAGACCCAGAAGGCCACCATCCTGGAGGCCAGCCTGAAGAAGCTGATCCCCGCATGGGAGTTCACGATTATCCCTTACAACGGCCAGAAGCACCAGAGCGACATCACCGACATCGTGTCCAGCCTGCAGCTGCAGTTCGAAAGCAGCGAGGAGGCCGACAAGGGGAATAGCCACAGCAAGAAGATGCTGAAGGCCCTGCTGTCCGAAGGCGAGAGCATCTGGGAGATTACCGAGAAGATCCTGAACAGCTTCGAGTACACCAGCAGATTTACCAAAACGAAGACCCTGTACCAGTTCCTGTTCCTGGCCACATTCATCAACTGCGGCAGGTTCAGCGACATCAAGAACGTGGACCCGAAGAGCTTCAAGCTCGTCCAGAACAAGTATCTGGGCGTGATCATTCAGTGCCTGGTCACGGAGACCAAGACAAGCGTGTCCAGGCACATCTACTTTTTCAGCGCCAGAGGCAGGATCGACCCCCTGGTGTACCTGGACGAGTTCCTGAGGAACAGCGAGCCCGTGCTGAAGAGAGTGAACAGGACCGGCAACAGCAGCAGCAACAAGCAGGAGTACCAGCTGCTGAAGGACAACCTGGTGCGCAGCTACAACAAGGCCCTGAAGAAGAACGCCCCCTACCCCATCTTCGCTATTAAAAACGGCCCTAAGAGCCACATCGGCAGGCACCTGATGACCAGCTTTCTGAGCATGAAGGGCCTGACCGAGCTGACAAACGTGGTGGGCAACTGGAGCGACAAGAGGGCCTCCGCCGTGGCCAGGACCACCTACACCCACCAGATCACCGCCATCCCCGACCACTACTTCGCCCTGGTGTCCAGGTACTACGCCTACGACCCCATCAGTAAGGAGATGATCGCCCTGAAGGACGAGACCAACCCCATCGAGGAGTGGCAGCACATCGAGCAGCTGAAGGGCAGCGCCGAGGGCAGCATCAGATACCCCGCCTGGAACGGCATTATAAGCCAGGAGGTGCTGGACTACCTGAGCAGCTACATCAACAGGCGGATCTGAAAGAGGTCGCTGCTGAGAAGACCCCTGGCAGCTCCCGAGCTAGCAGTGAATTTGTCGCTCTCCCTCATTTCCCAATGCTTGCCCTCTTGTCTCCCTCTTATCAGGCCTAGGGCAGGAGTGGGTCAGGAGGAAGGTTGCTTGGTGACTCGGGTCTCGGTGGCCTGTTTTGGTGCAATCCCAGTGAACAGTGACACTCTCGAAGTACAGGAGCATCTGGAGACACCTCCGGGCCCTTCTG

*Slco2a1-P2A-Cre*:

TGCCCCTGGGCCTCACCATACCTGTCTCTTCCTGCCTCATAGGTACCTGGGCCTACAGGTAATCTACAAGGTCTTGGGCACACTGCTGCTCTTCTTCATCAGCTGGAGGGTGAAGAAGAACAGGGAATACAGTCTGCAGGAGAATGCTTCCGGATTGATTGGAAGCGGAGCTACTAACTTCTCCCTGTTGAAACAAGCAGGGGATGTCGAAGAGAATCCTGGACCTATGGCTCCTAAGAAGAAGAGGAAGGTGATGAGCCAGTTCGACATCCTGTGCAAGACTCCTCCAAAGGTGCTGGTGCGGCAGTTCGTGGAGAGATTCGAGAGGCCCAGCGGCGAGAAGATCGCCAGCTGTGCCGCCGAGCTGACCTACCTGTGCTGGATGATCACCCACAACGGCACCGCCATCAAGAGGGCCACCTTCATGAGCTACAACACCATCATCAGCAACAGCCTGAGCTTCGACATCGTGAACAAGAGCCTGCAGTTCAAGTACAAGACCCAGAAGGCCACCATCCTGGAGGCCAGCCTGAAGAAGCTGATCCCCGCCTGGGAGTTCACCATCATCCCTTACAACGGCCAGAAGCACCAGAGCGACATCACCGACATCGTGTCCAGCCTGCAGCTGCAGTTCGAGAGCAGCGAGGAGGCCGACAAGGGCAACAGCCACAGCAAGAAGATGCTGAAGGCCCTGCTGTCCGAGGGCGAGAGCATCTGGGAGATCACCGAGAAGATCCTGAACAGCTTCGAGTACACCAGCAGGTTCACCAAGACCAAGACCCTGTACCAGTTCCTGTTCCTGGCCACATTCATCAACTGCGGCAGGTTCAGCGACATCAAGAACGTGGACCCCAAGAGCTTCAAGCTGGTGCAGAACAAGTACCTGGGCGTGATCATTCAGTGCCTGGTGACCGAGACCAAGACAAGCGTGTCCAGGCACATCTACTTTTTCAGCGCCAGAGGCAGGATCGACCCCCTGGTGTACCTGGACGAGTTCCTGAGGAACAGCGAGCCCGTGCTGAAGAGAGTGAACAGGACCGGCAACAGCAGCAGCAACAAGCAGGAGTACCAGCTGCTGAAGGACAACCTGGTGCGCAGCTACAACAAGGCCCTGAAGAAGAACGCCCCCTACCCCATCTTCGCTATCAAGAACGGCCCTAAGAGCCACATCGGCAGGCACCTGATGACCAGCTTTCTGAGCATGAAGGGCCTGACCGAGCTGACAAACGTGGTGGGCAACTGGAGCGACAAGAGGGCCTCCGCCGTGGCCAGGACCACCTACACCCACCAGATCACCGCCATCCCCGACCACTACTTCGCCCTGGTGTCCAGGTACTACGCCTACGACCCCATCAGCAAGGAGATGATCGCCCTGAAGGACGAGACCAACCCCATCGAGGAGTGGCAGCACATCGAGCAGCTGAAGGGCAGCGCCGAGGGCAGCATCAGATACCCCGCCTGGAACGGCATCATCAGCCAGGAGGTGCTGGACTACCTGAGCAGCTACATCAACAGGCGGATCTGACCTTCAGCTGGGACTACTGCCCTGCCCCAGAGACTGGATATCCTACCCCTCCACACCTACCTATATTAACTAATGTTAGCATGCCTTCCTCCTCCTTCC

Transfected cells were cultured and resulting colonies directly screened by PCR for correct integration using the following genotyping primers: flanking primer ATGCATTGCTTCATGCCATA and internal recombinase primer CCTTCAGCAGCTGGTACTCC for *Npnt-P2A-FlpO* left homology arm; GATTGAGGTCAGGCCAGAAG and TCGACATCGTGAACAAGAGC for *Npnt-P2A-FlpO* right homology arm; CTGGTGAAAGGGGAACTCTTGCT and GATCCCTGAACATGTCCATCAGG for *Slco2a1-P2A-Cre* left homology arm; TACAGCATCCCTGACAAACACCA and TAGCACCGCAGGTGTAGAGAAGG for *Slco2a1-P2A-Cre* right homology arm.

The inserted transgenes were fully sequenced and candidate lines were analysed for normal karyotype. Lines passing quality control were aggregated with albino morulae and implanted into pseudopregnant females, producing germline-competent chimeric founders which in turn were crossed with the appropriate reporter lines on the C57/BL6 background.

### Ethics oversight

All experimental procedures using live animals were performed according to protocols approved by Institutional Animal Care and Use Committees (IACUC) of all participating institutions: Allen Institute for Brain Science, Baylor College of Medicine, Broad Institute of MIT and Harvard, Cold Spring Harbor Laboratory, Harvard University, Salk Institute for Biological Studies, University of California Berkeley, University of California San Diego and University of Southern California. Macaque experiments were performed on animals designated for euthanasia via the Washington National Primate Research Center’s Tissue Distribution Program.

Postmortem adult human brain tissue collection was performed in accordance with the provisions of the United States Uniform Anatomical Gift Act of 2006 described in the California Health and Safety Code section 7150 (effective 1 January 2008) and other applicable state and federal laws and regulations. The Western Institutional Review Board reviewed tissue collection processes and determined that they did not constitute human subjects research requiring institutional review board (IRB) review. Before commencing the human Patch-seq, the donor provided informed consent and experimental procedures were approved by the hospital institute review board.

### Reporting summary

Further information on research design is available in the Nature Research Reporting Summary linked to this paper.

## Online content

Any methods, additional references, Nature Research reporting summaries, source data, extended data, supplementary information, acknowledgements, peer review information; details of author contributions and competing interests; and statements of data and code availability are available at 10.1038/s41586-021-03950-0.

### Supplementary information

Reporting Summary

Supplementary Table 1 Confusion matrices corresponding to Extended Data Figure 5.

Supplementary Table 2 Cell type nomenclature.

Peer Review File

## Figures and Tables

**Extended Data Table 1:**
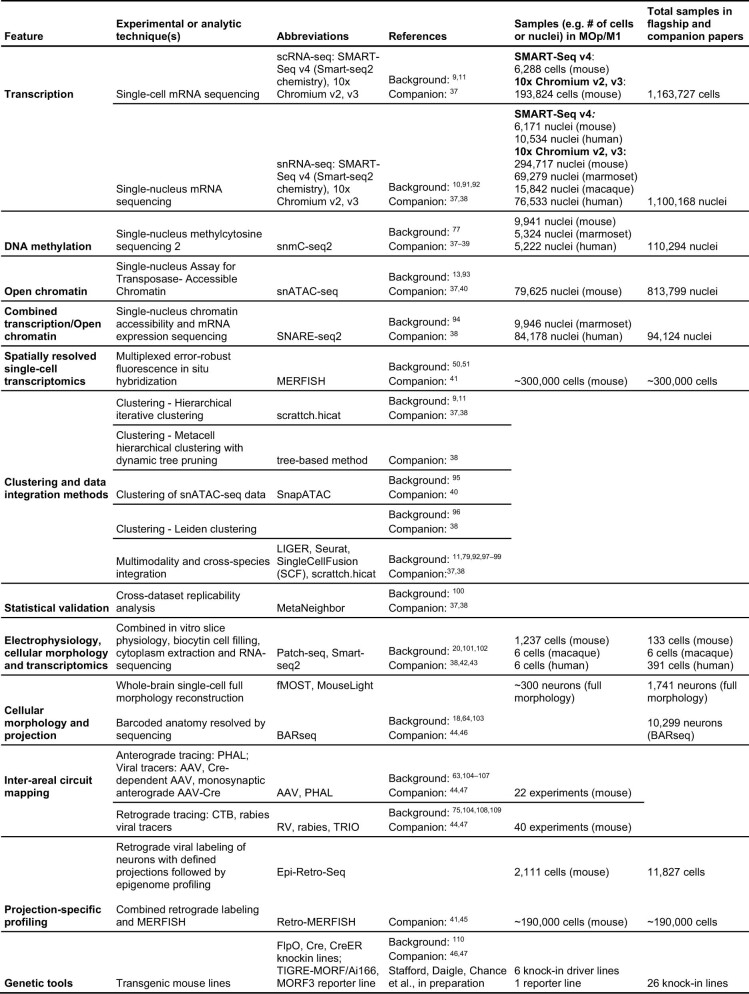
Experimental and computational techniques used in this study and associated datasets^91–110^

**Extended Data Table 2:**
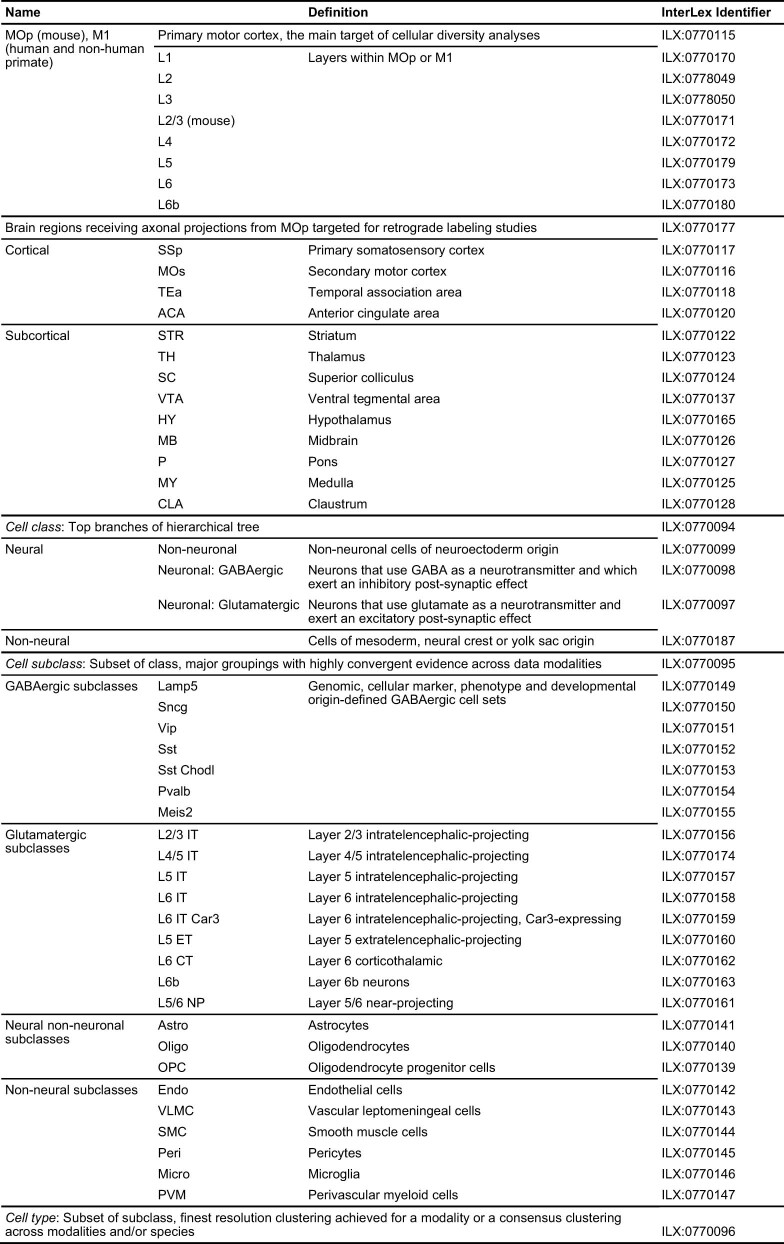
Glossary

## Data Availability

Primary data are accessible through the Brain Cell Data Center and data archives. Brain Cell Data Center (BCDC), Overall BICCN organization and data, www.biccn.org. Neuroscience Multi-omic Data Archive (NeMO), RRID:SCR_016152. Brain Image Library (BIL), RRID:SCR_017272. Distributed Archives for Neurophysiology Data Integration (DANDI), RRID:SCR_017571. Publicly used databases in study: NCBI Homologene, 11/22/2019, https://www.ncbi.nlm.nih.gov/homologene, GENCODE mm10 (v16), https://www.gencodegenes.org, JASPAR 2020 database, http://jaspar.genereg.net. All data resources associated with this publication are available as listed at: https://github.com/BICCN/CellCensusMotorCortex and 10.5281/zenodo.4726182.

## Code availability

All code and libraries used in the manuscript are available at https://github.com/BICCN/CellCensusMotorCortex and 10.5281/zenodo.4726182.

## References

[1] Somogyi, P. & Klausberger, T. Defined types of cortical interneurone structure space and spike timing in the hippocampus. *J. Physiol.* **562**, 9–26 (2005). 15539390 10.1113/jphysiol.2004.078915 PMC1665488

[2] Sanes, J. R. & Masland, R. H. The types of retinal ganglion cells: current status and implications for neuronal classification. *Annu. Rev. Neurosci.* **38**, 221–246 (2015). 25897874 10.1146/annurev-neuro-071714-034120

[3] Zeng, H. & Sanes, J. R. Neuronal cell-type classification: challenges, opportunities and the path forward. *Nat. Rev. Neurosci.* **18**, 530–546 (2017). 28775344 10.1038/nrn.2017.85

[4] Huang, Z. J. & Paul, A. The diversity of GABAergic neurons and neural communication elements. *Nat. Rev. Neurosci.* **20**, 563–572 (2019). 31222186 10.1038/s41583-019-0195-4 PMC8796706

[5] Mukamel, E. A. & Ngai, J. Perspectives on defining cell types in the brain. *Curr. Opin. Neurobiol.* **56**, 61–68 (2019). 30530112 10.1016/j.conb.2018.11.007 PMC6551297

[6] Petilla Interneuron Nomenclature Group. Petilla terminology: nomenclature of features of GABAergic interneurons of the cerebral cortex. *Nat. Rev. Neurosci.* **9**, 557–568 (2008). 18568015 10.1038/nrn2402 PMC2868386

[7] Zeisel, A. et al. Molecular architecture of the mouse nervous system. *Cell* **174**, 999–1014.e22 (2018). 30096314 10.1016/j.cell.2018.06.021 PMC6086934

[8] Saunders, A. et al. Molecular diversity and specializations among the cells of the adult mouse brain. *Cell* **174**, 1015–1030.e16 (2018). 30096299 10.1016/j.cell.2018.07.028 PMC6447408

[9] Tasic, B. et al. Shared and distinct transcriptomic cell types across neocortical areas. *Nature* **563**, 72–78 (2018). 30382198 10.1038/s41586-018-0654-5 PMC6456269

[10] Hodge, R. D. et al. Conserved cell types with divergent features in human versus mouse cortex. *Nature* **573**, 61–68 (2019). 31435019 10.1038/s41586-019-1506-7 PMC6919571

[11] Yao, Z. et al. A taxonomy of transcriptomic cell types across the isocortex and hippocampal formation. *Cell* **184**, 3222–3241.e26 (2021). 34004146 10.1016/j.cell.2021.04.021 PMC8195859

[12] Luo, C. et al. Single-cell methylomes identify neuronal subtypes and regulatory elements in mammalian cortex. *Science* **357**, 600–604 (2017). 28798132 10.1126/science.aan3351 PMC5570439

[13] Preissl, S. et al. Single-nucleus analysis of accessible chromatin in developing mouse forebrain reveals cell-type-specific transcriptional regulation. *Nat. Neurosci.* **21**, 432–439 (2018). 29434377 10.1038/s41593-018-0079-3 PMC5862073

[14] Lake, B. B. et al. Integrative single-cell analysis of transcriptional and epigenetic states in the human adult brain. *Nat. Biotechnol.* **36**, 70–80 (2018). 29227469 10.1038/nbt.4038 PMC5951394

[15] Cusanovich, D. A. et al. A single-cell atlas of in vivo mammalian chromatin accessibility. *Cell* **174**, 1309–1324.e18 (2018). 30078704 10.1016/j.cell.2018.06.052 PMC6158300

[16] Armand, E. J., Li, J., Xie, F., Luo, C. & Mukamel, E. A. Single-cell sequencing of brain cell transcriptomes and epigenomes. *Neuron* **109**, 11–26 (2021). 33412093 10.1016/j.neuron.2020.12.010 PMC7808568

[17] Yuste, R. et al. A community-based transcriptomics classification and nomenclature of neocortical cell types. *Nat. Neurosci.* **23**, 1456–1468 (2020). 32839617 10.1038/s41593-020-0685-8 PMC7683348

[18] Winnubst, J. et al. Reconstruction of 1,000 projection neurons reveals new cell types and organization of long-range connectivity in the mouse brain. *Cell* **179**, 268–281.e13 (2019). 31495573 10.1016/j.cell.2019.07.042 PMC6754285

[19] Zhong, Q. et al. High-definition imaging using line-illumination modulation microscopy. *Nat. Methods* **18**, 309–315 (2021). 33649587 10.1038/s41592-021-01074-x

[20] Cadwell, C. R. et al. Electrophysiological, transcriptomic and morphologic profiling of single neurons using Patch-seq. *Nat. Biotechnol.* **34**, 199–203 (2016). 26689543 10.1038/nbt.3445 PMC4840019

[21] Fuzik, J. et al. Integration of electrophysiological recordings with single-cell RNA-seq data identifies neuronal subtypes. *Nat. Biotechnol.* **34**, 175–183 (2016). 26689544 10.1038/nbt.3443 PMC4745137

[22] Lein, E., Borm, L. E. & Linnarsson, S. The promise of spatial transcriptomics for neuroscience in the era of molecular cell typing. *Science* **358**, 64–69 (2017). 28983044 10.1126/science.aan6827

[23] Zhuang, X. Spatially resolved single-cell genomics and transcriptomics by imaging. *Nat. Methods* **18**, 18–22 (2021). 33408406 10.1038/s41592-020-01037-8 PMC9805800

[24] Close, J. L., Long, B. R. & Zeng, H. Spatially resolved transcriptomics in neuroscience. *Nat. Methods* **18**, 23–25 (2021). 33408398 10.1038/s41592-020-01040-z

[25] Huang, Z. J. & Zeng, H. Genetic approaches to neural circuits in the mouse. *Annu. Rev. Neurosci.* **36**, 183–215 (2013). 23682658 10.1146/annurev-neuro-062012-170307

[26] Daigle, T. L. et al. A suite of transgenic driver and reporter mouse lines with enhanced brain-cell-type targeting and functionality. *Cell* **174**, 465–480.e22 (2018). 30007418 10.1016/j.cell.2018.06.035 PMC6086366

[27] He, M. et al. Strategies and tools for combinatorial targeting of GABAergic neurons in mouse cerebral cortex. *Neuron* **91**, 1228–1243 (2016). 27618674 10.1016/j.neuron.2016.08.021 PMC5223593

[28] Dimidschstein, J. et al. A viral strategy for targeting and manipulating interneurons across vertebrate species. *Nat. Neurosci.* **19**, 1743–1749 (2016). 27798629 10.1038/nn.4430 PMC5348112

[29] Vormstein-Schneider, D. et al. Viral manipulation of functionally distinct interneurons in mice, non-human primates and humans. *Nat. Neurosci.* **23**, 1629–1636 (2020). 32807948 10.1038/s41593-020-0692-9 PMC8015416

[30] Graybuck, L. T. et al. Enhancer viruses for combinatorial cell-subclass-specific labeling. *Neuron* **109**, 1449–1464.e13 (2021). 33789083 10.1016/j.neuron.2021.03.011 PMC8610077

[31] Hrvatin, S. et al. A scalable platform for the development of cell-type-specific viral drivers. *eLife* **8**, e48089 (2019). 31545165 10.7554/eLife.48089 PMC6776442

[32] Mich, J. K. et al. Functional enhancer elements drive subclass-selective expression from mouse to primate neocortex. *Cell Rep.* **34**, 108754 (2021). 33789096 10.1016/j.celrep.2021.108754 PMC8163032

[33] Ecker, J. R. et al. The BRAIN initiative cell census consortium: lessons learned toward generating a comprehensive brain cell atlas. *Neuron* **96**, 542–557 (2017). 29096072 10.1016/j.neuron.2017.10.007 PMC5689454

[34] Wang, Q. et al. The Allen Mouse Brain Common Coordinate Framework: a 3D reference atlas. *Cell* **181**, 936–953.e20 (2020). 32386544 10.1016/j.cell.2020.04.007 PMC8152789

[35] Lemon, R. N. Descending pathways in motor control. *Annu. Rev. Neurosci.* **31**, 195–218 (2008). 18558853 10.1146/annurev.neuro.31.060407.125547

[36] Svoboda, K. & Li, N. Neural mechanisms of movement planning: motor cortex and beyond. *Curr. Opin. Neurobiol.* **49**, 33–41 (2018). 29172091 10.1016/j.conb.2017.10.023

[37] Yao, Z. et al. An integrated transcriptomic and epigenomic atlas of mouse primary motor cortex cell types. Preprint at 10.1101/2020.02.29.970558 (2020).

[38] Bakken, T. E. et al. Evolution of cellular diversity in primary motor cortex of human, marmoset monkey, and mouse. Preprint at 10.1101/2020.03.31.016972 (2020).

[39] Liu, H. et al. DNA methylation atlas of the mouse brain at single-cell resolution. Preprint at 10.1101/2020.04.30.069377 (2020). 10.1038/s41586-020-03182-8 PMC8494641 34616061

[40] Li, Y. E. et al. An atlas of gene regulatory elements in adult mouse cerebrum. Preprint at 10.1101/2020.05.10.087585 (2020). 10.1038/s41586-021-03604-1 PMC8494637 34616068

[41] Zhang, M. et al. Molecular, spatial and projection diversity of neurons in primary motor cortex revealed by in situ single-cell transcriptomics. Preprint at 10.1101/2020.06.04.105700 (2020).

[42] Scala, F. et al. Phenotypic variation of transcriptomic cell types in mouse motor cortex. *Nature*10.1038/s41586-020-2907-3 (2020). 10.1038/s41586-020-2907-3 PMC8113357 33184512

[43] Berg, J. et al. Human cortical expansion involves diversification and specialization of supragranular intratelencephalic-projecting neurons. Preprint at 10.1101/2020.03.31.018820 (2020).

[44] Muñoz-Castaneda, R. et al. Cellular anatomy of the mouse primary motor cortex. Preprint at 10.1101/2020.10.02.323154 (2020). 10.1038/s41586-021-03970-w PMC8494646 34616071

[45] Zhang, Z. et al. Epigenomic diversity of cortical projection neurons in the mouse brain. Preprint at 10.1101/2020.04.01.019612 (2020). 10.1038/s41586-021-03223-w PMC8494636 34616065

[46] Peng, H. et al. Brain-wide single neuron reconstruction reveals morphological diversity in molecularly defined striatal, thalamic, cortical and claustral neuron types. Preprint at 10.1101/675280 (2020).

[47] Matho, K. S. et al. Genetic dissection of glutamatergic neuron subpopulations and developmental trajectories in the cerebral cortex. Preprint at 10.1101/2020.04.22.054064 (2020).

[48] Harris, K. D. & Shepherd, G. M. G. The neocortical circuit: themes and variations. *Nat. Neurosci.* **18**, 170–181 (2015). 25622573 10.1038/nn.3917 PMC4889215

[49] Molyneaux, B. J., Arlotta, P., Menezes, J. R. L. & Macklis, J. D. Neuronal subtype specification in the cerebral cortex. *Nat. Rev. Neurosci.* **8**, 427–437 (2007). 17514196 10.1038/nrn2151

[50] Chen, K. H., Boettiger, A. N., Moffitt, J. R., Wang, S. & Zhuang, X. Spatially resolved, highly multiplexed RNA profiling in single cells. *Science* **348**, aaa6090 (2015). 25858977 10.1126/science.aaa6090 PMC4662681

[51] Moffitt, J. R. et al. Molecular, spatial, and functional single-cell profiling of the hypothalamic preoptic region. *Science* **362**, eaau5324 (2018). 30385464 10.1126/science.aau5324 PMC6482113

[52] Scheibel, M. E., Davies, T. L., Lindsay, R. D. & Scheibel, A. B. Basilar dendrite bundles of giant pyramidal cells. *Exp. Neurol.* **42**, 307–319 (1974). 4207597 10.1016/0014-4886(74)90028-4

[53] Economo, M. N. et al. Distinct descending motor cortex pathways and their roles in movement. *Nature* **563**, 79–84 (2018). 30382200 10.1038/s41586-018-0642-9

[54] Bouyain, S. & Watkins, D. J. The protein tyrosine phosphatases PTPRZ and PTPRG bind to distinct members of the contactin family of neural recognition molecules. *Proc. Natl. Acad. Sci. USA* **107**, 2443–2448 (2010). 20133774 10.1073/pnas.0911235107 PMC2823867

[55] Greig, L. C., Woodworth, M. B., Galazo, M. J., Padmanabhan, H. & Macklis, J. D. Molecular logic of neocortical projection neuron specification, development and diversity. *Nat. Rev. Neurosci.* **14**, 755–769 (2013). 24105342 10.1038/nrn3586 PMC3876965

[56] Di Bella, D. J. et al. Molecular logic of cellular diversification in the mammalian cerebral cortex. Preprint at 10.1101/2020.07.02.185439 (2020).

[57] Chou, S.-J. & Tole, S. Lhx2, an evolutionarily conserved, multifunctional regulator of forebrain development. *Brain Res.* **1705**, 1–14 (2019). 29522720 10.1016/j.brainres.2018.02.046

[58] Englund, C. et al. Pax6, Tbr2, and Tbr1 are expressed sequentially by radial glia, intermediate progenitor cells, and postmitotic neurons in developing neocortex. *J. Neurosci.* **25**, 247–251 (2005). 15634788 10.1523/JNEUROSCI.2899-04.2005 PMC6725189

[59] Muralidharan, B. et al. LHX2 interacts with the NuRD complex and regulates cortical neuron subtype determinants *Fezf2* and *Sox11*. *J. Neurosci.* **37**, 194–203 (2017). 28053041 10.1523/JNEUROSCI.2836-16.2016 PMC5214630

[60] Eckler, M. J. et al. Multiple conserved regulatory domains promote *Fezf2* expression in the developing cerebral cortex. *Neural Dev.* **9**, 6 (2014). 24618363 10.1186/1749-8104-9-6 PMC4008173

[61] Vasistha, N. A. et al. Cortical and clonal contribution of *Tbr2* expressing progenitors in the developing mouse brain. *Cereb. Cortex* **25**, 3290–3302 (2015). 24927931 10.1093/cercor/bhu125 PMC4585488

[62] Gerfen, C. R., Paletzki, R. & Heintz, N. GENSAT BAC cre-recombinase driver lines to study the functional organization of cerebral cortical and basal ganglia circuits. *Neuron* **80**, 1368–1383 (2013). 24360541 10.1016/j.neuron.2013.10.016 PMC3872013

[63] Harris, J. A. et al. Hierarchical organization of cortical and thalamic connectivity. *Nature* **575**, 195–202 (2019). 31666704 10.1038/s41586-019-1716-z PMC8433044

[64] Chen, X. et al. High-throughput mapping of long-range neuronal projection using in situ sequencing. *Cell* **179**, 772–786.e19 (2019). 31626774 10.1016/j.cell.2019.09.023 PMC7836778

[65] Yamawaki, N., Borges, K., Suter, B. A., Harris, K. D. & Shepherd, G. M. G. A genuine layer 4 in motor cortex with prototypical synaptic circuit connectivity. *eLife* **3**, e05422 (2014). 25525751 10.7554/eLife.05422 PMC4290446

[66] García-Cabezas, M. Á. & Barbas, H. Area 4 has layer IV in adult primates. *Eur. J. Neurosci.* **39**, 1824–1834 (2014). 24735460 10.1111/ejn.12585 PMC4201116

[67] Narayanan, R. T., Udvary, D. & Oberlaender, M. Cell type-specific structural organization of the six layers in rat barrel cortex. *Front. Neuroanat.* **11**, 91 (2017). 29081739 10.3389/fnana.2017.00091 PMC5645532

[68] Harris, K. D. et al. Classes and continua of hippocampal CA1 inhibitory neurons revealed by single-cell transcriptomics. *PLoS Biol.* **16**, e2006387 (2018). 29912866 10.1371/journal.pbio.2006387 PMC6029811

[69] Stanley, G., Gokce, O., Malenka, R. C., Südhof, T. C. & Quake, S. R. Continuous and discrete neuron types of the adult murine striatum. *Neuron* **105**, 688–699.e8 (2020). 31813651 10.1016/j.neuron.2019.11.004

[70] Arendt, D. et al. The origin and evolution of cell types. *Nat. Rev. Genet.* **17**, 744–757 (2016). 27818507 10.1038/nrg.2016.127

[71] Kobak, D. & Berens, P. The art of using *t*-SNE for single-cell transcriptomics. *Nat. Commun.* **10**, 5416 (2019). 31780648 10.1038/s41467-019-13056-x PMC6882829

[72] Saiki, A. et al. In vivo spiking dynamics of intra- and extratelencephalic projection neurons in rat motor cortex. *Cereb. Cortex* **28**, 1024–1038 (2018). 28137723 10.1093/cercor/bhx012

[73] Baker, A. et al. Specialized subpopulations of deep-layer pyramidal neurons in the neocortex: bridging cellular properties to functional consequences. *J. Neurosci.* **38**, 5441–5455 (2018). 29798890 10.1523/JNEUROSCI.0150-18.2018 PMC6001033

[74] Love, M. I., Huber, W. & Anders, S. Moderated estimation of fold change and dispersion for RNA-seq data with DESeq2. *Genome Biol.* **15**, 550 (2014). 25516281 10.1186/s13059-014-0550-8 PMC4302049

[75] Tervo, D. G. R. et al. A designer AAV variant permits efficient retrograde access to projection neurons. *Neuron* **92**, 372–382 (2016). 27720486 10.1016/j.neuron.2016.09.021 PMC5872824

[76] Mo, A. et al. Epigenomic signatures of neuronal diversity in the mammalian brain. *Neuron* **86**, 1369–1384 (2015). 26087164 10.1016/j.neuron.2015.05.018 PMC4499463

[77] Luo, C. et al. Robust single-cell DNA methylome profiling with snmC-seq2. *Nat. Commun.* **9**, 3824 (2018). 30237449 10.1038/s41467-018-06355-2 PMC6147798

[78] Wolf, F. A., Angerer, P. & Theis, F. J. SCANPY: large-scale single-cell gene expression data analysis. *Genome Biol.* **19**, 15 (2018). 29409532 10.1186/s13059-017-1382-0 PMC5802054

[79] Luo, C. et al. Single nucleus multi-omics links human cortical cell regulatory genome diversity to disease risk variants. Preprint at 10.1101/2019.12.11.873398 (2019).

[80] Hie, B., Bryson, B. & Berger, B. Efficient integration of heterogeneous single-cell transcriptomes using Scanorama. *Nat. Biotechnol.* **37**, 685–691 (2019). 31061482 10.1038/s41587-019-0113-3 PMC6551256

[81] Zhang, Y. et al. Model-based analysis of ChIP-seq (MACS). *Genome Biol.* **9**, R137 (2008). 18798982 10.1186/gb-2008-9-9-r137 PMC2592715

[82] Corces, M. R. et al. The chromatin accessibility landscape of primary human cancers. *Science* **362**, (2018). 10.1126/science.aav1898 PMC6408149 30361341

[83] Pliner, H. A. et al. Cicero predicts *cis*-regulatory DNA interactions from single-cell chromatin accessibility data. *Mol. Cell* **71**, 858–871.e8 (2018). 30078726 10.1016/j.molcel.2018.06.044 PMC6582963

[84] Delignette-Muller, M. & Dutang, C. fitdistrplus: an R package for fitting distributions. *J. Stat. Softw.* **64**, 1–34 (2015).

[85] Hoyer, P. O. Non-negative matrix factorization with sparseness constraints. *J. Mach. Learn. Res.* **5**, 1457–1469 (2004).

[86] Fornes, O. et al. JASPAR 2020: update of the open-access database of transcription factor binding profiles. *Nucleic Acids Res.* **48**, D87–D92 (2020). 31701148 10.1093/nar/gkz1001 PMC7145627

[87] McLeay, R. C. & Bailey, T. L. Motif enrichment analysis: a unified framework and an evaluation on ChIP data. *BMC Bioinformatics* **11**, 165 (2010). 20356413 10.1186/1471-2105-11-165 PMC2868005

[88] Claudi, F., Tyson, A. L. & Branco, T. Brainrender. A Python based software for visualisation of neuroanatomical and morphological data. Preprint at 10.1101/2020.02.23.961748 (2020).

[89] Yin, L. et al. Epigenetic regulation of neuronal cell specification inferred with single cell ‘Omics’ data. *Comput. Struct. Biotechnol. J.* **18**, 942–952 (2020). 32368329 10.1016/j.csbj.2020.04.007 PMC7184133

[90] Harrington, A. J. et al. MEF2C regulates cortical inhibitory and excitatory synapses and behaviors relevant to neurodevelopmental disorders. *eLife* **5**, (2016). 10.7554/eLife.20059 PMC5094851 27779093

[91] Kozareva, V. et al. A transcriptomic atlas of the mouse cerebellum reveals regional specializations and novel cell types. Preprint at 10.1101/2020.03.04.976407 (2020).

[92] Krienen, F. M. et al. Innovations in primate interneuron repertoire. *Nature* **586**, 262–269 (2020). 32999462 10.1038/s41586-020-2781-z PMC7957574

[93] Cusanovich, D. A. et al. Multiplex single cell profiling of chromatin accessibility by combinatorial cellular indexing. *Science* **348**, 910–914 (2015). 25953818 10.1126/science.aab1601 PMC4836442

[94] Chen, S., Lake, B. B. & Zhang, K. High-throughput sequencing of the transcriptome and chromatin accessibility in the same cell. *Nat. Biotechnol.* **37**, 1452–1457 (2019). 31611697 10.1038/s41587-019-0290-0 PMC6893138

[95] Feng, R. et al. Comprehensive analysis of single cell ATAC-seq data with SnapATAC. *Nat. Commun.* **12**, 1337 (2021). 10.1038/s41467-021-21583-9 PMC7910485 33637727

[96] Traag, V. A., Waltman, L. & van Eck, N. J. From Louvain to Leiden: guaranteeing well-connected communities. *Sci. Rep.* **9**, 5233 (2019). 30914743 10.1038/s41598-019-41695-z PMC6435756

[97] Welch, J. D. et al. Single-cell multi-omic integration compares and contrasts features of brain cell identity. *Cell* **177**, 1873–1887.e17 (2019). 31178122 10.1016/j.cell.2019.05.006 PMC6716797

[98] Haghverdi, L., Lun, A. T. L., Morgan, M. D. & Marioni, J. C. Batch effects in single-cell RNA-sequencing data are corrected by matching mutual nearest neighbors. *Nat. Biotechnol.* **36**, 421–427 (2018). 29608177 10.1038/nbt.4091 PMC6152897

[99] Stuart, T. et al. Comprehensive integration of single-cell data. *Cell* **177**, 1888–1902.e21 (2019). 31178118 10.1016/j.cell.2019.05.031 PMC6687398

[100] Crow, M., Paul, A., Ballouz, S., Huang, Z. J. & Gillis, J. Characterizing the replicability of cell types defined by single cell RNA-sequencing data using MetaNeighbor. *Nat. Commun.* **9**, 884 (2018). 29491377 10.1038/s41467-018-03282-0 PMC5830442

[101] Cadwell, C. R. et al. Multimodal profiling of single-cell morphology, electrophysiology, and gene expression using Patch-seq. *Nat. Protoc.* **12**, 2531–2553 (2017). 29189773 10.1038/nprot.2017.120 PMC6422019

[102] Gouwens, N. W. et al. Integrated morphoelectric and transcriptomic classification of cortical GABAergic cells. *Cell* **183**, 935–953.e19 (2020). 33186530 10.1016/j.cell.2020.09.057 PMC7781065

[103] Gong, H. et al. High-throughput dual-colour precision imaging for brain-wide connectome with cytoarchitectonic landmarks at the cellular level. *Nat. Commun.* **7**, 12142 (2016). 27374071 10.1038/ncomms12142 PMC4932192

[104] Zingg, B. et al. Neural networks of the mouse neocortex. *Cell* **156**, 1096–1111 (2014). 24581503 10.1016/j.cell.2014.02.023 PMC4169118

[105] Zingg, B. et al. AAV-mediated anterograde transsynaptic tagging: mapping corticocollicular input-defined neural pathways for defense behaviors. *Neuron* **93**, 33–47 (2017). 27989459 10.1016/j.neuron.2016.11.045 PMC5538794

[106] Hintiryan, H. et al. The mouse cortico-striatal projectome. *Nat. Neurosci.* **19**, 1100–1114 (2016). 27322419 10.1038/nn.4332 PMC5564682

[107] Oh, S. W. et al. A mesoscale connectome of the mouse brain. *Nature* **508**, 207–214 (2014). 24695228 10.1038/nature13186 PMC5102064

[108] Reardon, T. R. et al. Rabies virus CVS-N2c(ΔG) strain enhances retrograde synaptic transfer and neuronal viability. *Neuron* **89**, 711–724 (2016). 26804990 10.1016/j.neuron.2016.01.004 PMC4760870

[109] Wickersham, I. R. et al. Monosynaptic restriction of transsynaptic tracing from single, genetically targeted neurons. *Neuron* **53**, 639–647 (2007). 17329205 10.1016/j.neuron.2007.01.033 PMC2629495

[110] Veldman, M. B. et al. Brainwide genetic sparse cell labeling to illuminate the morphology of neurons and glia with cre-dependent MORF mice. *Neuron* **108**, 111–127.e6 (2020). 32795398 10.1016/j.neuron.2020.07.019 PMC7572760

