# Supplementary material for: A multimodal cell census and atlas of the mammalian primary motor cortex
Source: Nature. 2021 Oct 6;598(7879):86–102. doi: 10.1038/s41586-021-03950-0 (PMC8494634; doi:10.1038/s41586-021-03950-0)
Supplement: Supplementary file 1 — Reporting Summary [file 41586_2021_3950_MOESM1_ESM.pdf]

nature research

Corresponding author(s): Edward M. Callaway, Hong-Wei Dong, Joseph R. Ecker, Mike Hawrylycz, Z. Josh Huang, Ed S. Lein, John Ngai, Pavel Osten, Bing Ren, Andreas Savas Tolias, Owen White, Hongkui Zeng, Xiaowei Zhuang

Last updated by author(s): July 22, 2021

Reporting Summary

Nature Research wishes to improve the reproducibility of the work that we publish. This form provides structure for consistency and transparency in reporting. For further information on Nature Research policies, see our Editorial Policies and the Editorial Policy Checklist.

Statistics

For all statistical analyses, confirm that the following items are present in the figure legend, table legend, main text, or Methods section.

n/a	Confirmed	
☐	☒	The exact sample size (*n*) for each experimental group/condition, given as a discrete number and unit of measurement
☐	☒	A statement on whether measurements were taken from distinct samples or whether the same sample was measured repeatedly
☐	☒	The statistical test(s) used AND whether they are one- or two-sided<br>*Only common tests should be described solely by name; describe more complex techniques in the Methods section.*
☐	☒	A description of all covariates tested
☐	☒	A description of any assumptions or corrections, such as tests of normality and adjustment for multiple comparisons
☐	☒	A full description of the statistical parameters including central tendency (e.g. means) or other basic estimates (e.g. regression coefficient) AND variation (e.g. standard deviation) or associated estimates of uncertainty (e.g. confidence intervals)
☐	☒	For null hypothesis testing, the test statistic (e.g. *F*, *t*, *r*) with confidence intervals, effect sizes, degrees of freedom and *P* value noted<br>*Give P values as exact values whenever suitable.*
☐	☒	For Bayesian analysis, information on the choice of priors and Markov chain Monte Carlo settings
☐	☒	For hierarchical and complex designs, identification of the appropriate level for tests and full reporting of outcomes
☐	☒	Estimates of effect sizes (e.g. Cohen's *d*, Pearson's *r*), indicating how they were calculated

Our web collection on statistics for biologists contains articles on many of the points above.

Software and code

Policy information about availability of computer code

Data collection

All data for this manuscript was collected in the set of corresponding core companion papers. Please refer to these papers for data generation and quantification software.

- An integrated transcriptomic and epigenomic atlas of mouse primary motor cortex cell types, Yao et al., 2021
- Evolution of cellular diversity in primary motor cortex of human, marmoset monkey, and mouse, Bakken et al., 2021
- Molecular, spatial and projection diversity of neurons in primary motor cortex revealed by in situ single-cell transcriptomics, Zhang et al, 2021
- Phenotypic variation of transcriptomic cell types in mouse motor cortex, Scala et al, 2020
- Cellular Anatomy of the Mouse Primary Motor Cortex, Munoz-Casteneda, 2021
- Genetic dissection of the glutamatergic neuron system in cerebral cortex, Matho et al,2021

- An atlas of gene regulatory elements in Adult Mouse Cerebrum, Li et al, 2021
- Human cortical expansion involves diversification and specialization of supragranular intratelencephalic-projecting neurons, Berg et al, 2021
- Brain-wide single neuron reconstruction reveals morphological diversity in molecularly defined striatal, thalamic, cortical and claustral neuron types, Peng et al, 2021
- DNA Methylation Atlas of the Mouse Brain at Single-Cell Resolution, Liu et al., 2021
- Epigenomic Diversity of Cortical Projection Neurons in the Mouse Brain, Zhang et al., 2021

Data analysis

Human, macaque, marmoset, mouse transcriptomics: Code for generating Figure 1b-h, ED Figure 2, and Figure 3h is available at http://data.nemoarchive.org/biccn/lab/lein/2020_M1_study_analysis/Transcriptomics/flagship/. Analysis was performed in RStudio using R version 3.5.3, R packages: Seurat 3.1.1, ggplot2 3.2.1, scrattch.hicat 0.0.22.

Estimation of cell type homology: A final dendrogram of consensus cell types was constructed by transforming the raw UMI counts to log2 CPM normalized counts. Up to 50 marker genes per cross-species cluster were identified by using the scrattch.hicat (v0.0.22) (https://github.com/AllenInstitute/scrattch.hicat) display_cl and select_markers functions with the following parameters; q1.th = 0.4, q.diff.th = 0.5, de.score.th = 80.

Cross-species differential gene expression and correlations: To calculate the number of DE genes between each species pair for each cross-species cluster, we used a pseudobulk comparison method from DESeq2 (v1.30.0)99. For a given cross-species cluster, each sample was split by species and donor, then a Wald test was performed between each species pair.

Integration of L5 ET cells from Epi-Retro-Seq and 10x snRNA-Seq: Top 5,000 snRNA-seq highly variable genes were identified with Scanpy v1.8.1 and z-score scaled across all the cells. Top 5,000 Epi-Retro-Seq highly variable genes were identified with AllCools and z-score scaled across all the cells. The 1,512 genes as the intersection between the two highly variable gene lists were used in Scanorama v1.7.1 to integrate the z-scored expression matrix and minus z-scored methylation matrix with sigma equal to 100.

Integrating mouse transcriptomic, spatially resolved transcriptomic, and epigenomic datasets: The integrated clustering and embedding of the 11 datasets are then generated by projecting all datasets into the 10x v2 scRNA-seq dataset using SingleCellFusion. MERFISH data was analyzed using custom Python code. This code is available at https://github.com/ZhuangLab/MERlin.

Identification of candidate cis-regulatory elements: For peak calling in the snATAC-seq data, we extracted all the fragments for each cluster, and then performed peak calling on each aggregate profile using MACS2 v2.2.7.1. using Python 3.6.

Predicting enhancer-promoter interactions: First, co-accessible cCREs are identified for all open regions in all neurons types (cell clusters with less than 100 nuclei from snATAC-seq are excluded), using Cicero v1.0.0.

Identification of cis-regulatory modules: NMF (Python package: sklearn v0.24.2) was used to decompose the cell-by-cCRE matrix V (N×M, N rows: cCRE, M columns: cell clusters) into a coefficient matrix H (R×M, R rows: number of modules) and a basis matrix W (N×R), with a given rank R.

Code availability: All code and libraries used in the manuscript are available at https://github.com/BICCN/CellCensusMotorCortex.
DOI: 10.5281/zenodo.4726182.

Data

All data is freely available for public use, see also "Data availability" section of the main manuscript.
Primary data is accessible through the Brain Cell Data Center and data archives.

- Brain Cell Data Center (BCDC), Overall BICCN organization and data, www.biccn.org

BRAIN Initiative Data Archives for BICCN data

- Neuroscience Multi-omic Data Archive (NeMO), RRID:SCR_016152
- Brain Image Library (BIL), RRID:SCR_017272
- Distributed Archives for Neurophysiology Data Integration (DANDI), RRID:SCR_017571

Publicly used databases in study:

- NCBI Homologene, 11/22/2019, https://www.ncbi.nlm.nih.gov/homologene
- GENCODE mm10 (v16), https://www.gencodegenes.org
- JASPAR 2020 database, http://jaspar.genereg.net

Data Availability: In addition to the raw data available through the archives all figure specific data sets are available at:

https://github.com/BICCN/CellCensusMotorCortex. DOI: 10.5281/zenodo.4726182.

Field-specific reporting

Please select the one below that is the best fit for your research. If you are not sure, read the appropriate sections before making your selection.

☒ Life sciences ☐ Behavioural & social sciences ☐ Ecological, evolutionary & environmental sciences

For a reference copy of the document with all sections, see nature.com/documents/nr-reporting-summary-flat.pdf

Life sciences study design

All studies must disclose on these points even when the disclosure is negative.

Sample size

Sample sizes for all data types are provided in Extended Data Table 1 of the manuscript. Specifically:

2,111 cells generated by Epi-Retro-Seq and 9876 cells generated by snmC-seq2 were analyzed. 79,625 nuclei generated by snATAC-seq were used.
6439 cells generated by SMART-seq and 6300 were analyzied
176584 cells generated by 10X v3 and 94170 were analyzed
145748 cells generated by 10X v2 and 122641 were analyzed
6848 nuclei were generated by SMAR-seq v4 and 6278 were analyzed.
91071 nuclei were generated by 10X v2 and 76714 were analyzed.
215823 nuclei were generated by 10X v3 (by Macoksco's lab) and 178926 were analyzed. 90266 nuclei were generated by 10X v3(by Allen Institute for Brain Science) and 40555 were analyzed.

For Epi-Retro-Seq:384 nuclei from each projection from 2 male and 2 female mice (except the MOp-SSp projection from which 768 nuclei were assayed) were analyzed. The sample size was chosen based on preliminary results from pilot studies that on average >=50 labeled projection neurons can be successfully collected from MOp of each animal.

Human, macaque, marmoset, mouse transcriptomics: For high-throughput single nucleus genomic sequencing of primate tissue, sufficient nuclei were profiled to capture the rarest neuronal cell types observed in mouse motor cortex. For lower throughput assays, sufficient nuclei were profiled to characterize all neuronal subclasses and most cell types.

MERFISH: Two replicate animals were imaged under each condition. From the two replicate animals imaged for the identification and spatial mapping of cell types, a total of ~300,000 cells were imaged, which generated a sufficient number of single-cell profiles and gave sufficient statistics for the effect sizes of interest. From the two replicate animals imaged for projection target mapping, a total of ~190,000 cells were imaged, which gave sufficient statistics for the effect sizes of interest.

Mouse Patch-seq: Sampling strategy was determined using pre-existing knowledge of the transcriptional diversity of the mouse cortex (Tasic et al., 2018; Yao et al., 2020) and based also on the variability of morphological and electrophysiological types predicted by existing literature (Jiang et al., 2015, Gouwens et al. 2019, Scala et al., 2019).

Human Patch-seq: Sampling strategy was determined using preexisting knowledge about the size of primate L5 ET neurons.

Anatomy: The sample sizes for different injection methods with different tracers were specified in Methods sections as described for different laboratories. In general, representative cases presented in all figures were selected from a much larger data pool. Each of the injections was repeated in at least two cases for verification purposes, and only tracing data that was validated is reported.

Transgenic line characterization: 2-3 individual animals per genotype (note: valuation of expression patterns of genetically encoded reporters was qualitative).

Data exclusions

snmC-Seq2 and Epi-Retro-Seq:
Poor quality nuclei were excluded from clustering if they failed to meet the following pre-established quality control (QC) thresholds:
< 500,000 non-clonal reads
> 1% non-conversion rate

snATAC-seq:
No samples were excluded.
For analysis as pre-established only nuclei with > 1,000 reads/nucleus and transcriptional start site enrichment > 10 were selected.
Potential barcode collisions were excluded from analysis

SMART-seq v4 cells/nuclei:
Cells that met any one of the following pre-established criteria were removed: < 100,000 total reads, < 1,000 detected genes (with CPM > 0), < 75% of reads aligned to genome, or CG dinucleotide odds ratio > 0.5.

10X cells cells/nuclei:
Cells were first classified as neuron or non-neuronal cell types, and neuronal cells with more than 2000 detected genes and non-neuronal cells with more than 1000 detected genes were selected, excluding cells with doublet score greater than 0.3 as a pre-established criterion.

Human, macaque, marmoset, mouse transcriptomics:
The following criteria were pre-established. Nuclei belonging to low-quality, sex-specific, or donor-specific clusters were removed from analyses. Briefly:

Human RNA-seq (SMART-seq v4):
> 30% cDNA longer than 400 base pairs
> 500,000 reads aligned to exonic or intronic sequence
> 40% of total reads aligned
> 50% unique reads
> 0.7 TA nucleotide ratio

Human and Macaque RNA-seq (10x v3): Pre-establihed acceptance criteria were:

> 500 (non-neuronal nuclei) or > 1000 (neuronal nuclei) genes detected
< 0.3 doublet score

Marmoset RNA-seq (10x v3):
Cell barcodes were filtered to distinguish true nuclei barcodes from empty beads and PCR artifacts by assessing proportions of ribosomal and mitochondrial reads. Pre-established criteria include ratio of intronic/exonic reads (> 50% of intronic reads), library size (> 1000 UMIs) and sequencing efficiency (true cell barcodes have higher reads/UMI).

Mouse RNA-seq (SMART-seq v4 and 10x v3): Pre-established criteria for rejection included
< 100,000 total reads, < 1,000 detected genes (CPM > 0), < 75% of reads aligned to genome, or CG dinucleotide odds ratio > 0.5. Cells were classified into broad classes of excitatory, inhibitory, and non-neuronal based on known markers, and cells with ambiguous identities were removed as doublets.

MERFISH: No data was excluded from consideration. All images were included in the basic analysis.

Mouse Patch-seq:
Cells meeting any of the pre-established exclusion criteria described in the following were declared low quality and did not get a t-type assignment: cells with the highest correlation below 0.4 (78 cells); cells that would be assigned to non-neural t-types, presumably due to RNA contamination (14 cells); cells with the highest correlation less than 0.02 above the maximal correlation in one of the other two transcriptomic orders (5 cells). Four cells were assigned to an excitatory t-type, despite having clearly inhibitory firing, morphology, and/or soma depth location (such as L1). The most likely cause was RNA contamination from excitatory cells that are much more abundant in the mouse cortex. These four cells were excluded from all analyses and visualizations (as if they did not pass the transcriptomic quality control). In addition, one cell was likely located outside of MOp, based on the slice anatomy, and was excluded as well. For the electrophysiology, the cells were not recorded or included when seal resistance values were <1 GΩ before achieving whole-cell configuration and/or initial access resistance was >30 MΩ. Cells were excluded from morphological analysis when the staining quality did not match pre-established criteria for inclusion. Cells that showed low staining quality such as poor fill, excessive background, dendritic or axonal truncation were not reconstructed and not included in the dataset.

Human Patch-seq:
Patch-seq samples with a mapping confidence < 0.5 were excluded from analysis as a pre-established criterion.

Anatomy:
The best most representative injections were chosen for the analysis. The others were excluded due to off-targeting of the injection site, missing/damaged tissue, weak tracer labeling of the axons or high background, etc. These were pre-established criteria.

Transgenic line characterization:
Data were excluded from failed experiments - i.e., in cases where no signal was detected or tissue was damaged during processing as a pre-established criterion.

Replication

Epi-Retro-Seq:
At least 2 male and 2 female mice were injected with AAV-retro-Cre for each projection target. Male and female samples were pooled separately for nuclei preparation.

snmC-seq2:
Each dissected region has at least two replicates, each replicate was pooled from 6-30 animals separately for nuclei preparation and downstream analyses.

snATAC-seq:
Experiments were performed for 2 biological replicates for each dissected region

sc/snRNA-seq:
The number of animals used for profiling in replication for each platform listed below:
10X v2 cells: 3 male
10X v2 nuclei: 2 male, 1 female
10X v3 cells: 3 male, 3 female
10X v3 nuclei: 5 male, 7 female
SMART-seq cells: 28 male, 17 female
SMART-seq nuclei: 8 male, 2 female

Human, macaque, marmoset, mouse transcriptomics:
All species clusters were examined to ensure representation from multiple donors and both sexes. Specifically, clustering reproducibility was measured by performing clustering analysis 100 times using a randomly-selected 80% of nuclei. Similarly, all cross-species clusters were examined for representation from all three species and final cluster assignment was based on 100 iterations of clustering using 95% of nuclei. Replicated findings by profiling tissue from multiple donors from each species (human, marmoset, and mouse). Donor effects are reported as Extended Data Figures in the companion manuscript.

MERFISH:
Reported results were replicated from two animals under each condition. Reported results were replicated with replicates generating similar results.

Mouse Patch-seq:
The results of this study were not directly replicated. However, all the results were collected from multiple animals from multiple litters per wild-type and transgenic lines. For our mouse and macaque recordings we compiled data across different animals (4 macaques; 6 mice)

Human Patch-seq:
The results were not directly replicated, but when possible data were collected from multiple subjects. The human Patch-seq data was from a rare surgical case and so replication was not possible.

Anatomy:
This study focuses on characterizing inputs/outputs of the primary motor cortex upper limb area (MOp-ul) using different tracing methods. Each of tracer injections were repeated multiple times in different animals. While the best, most representative cases were chosen for inclusion in the analysis data set, the other injections served as validation cases, demonstrating the replicability and consistency of tracer labeling.

Transgenic line characterization:
Expression patterns of genetically encoded reporters are typically representative of 2-3 animals of the same genotype.

Randomization

There was no randomization performed as the study does not involve multiple study groups.

Human, macaque, marmoset, mouse transcriptomics:
All species specimens were controls and were therefore allocated into the same experimental group. Randomization was not used. To compare datasets across species, random nuclei from each cluster were chosen to downsample each species' dataset, and ensure approximately equal representation of cell types at the subclass level. Additionally, for heatmap visualizations (Figure 2g), up to 50 random nuclei from each subclass for each species were chosen.

Blinding

There was no blinding performed as the study does not involve multiple study groups.

Human, macaque, marmoset, mouse transcriptomics:
Human specimens were de-identified and assigned a unique numerical code. Knowledge of which sample came from which species was necessary for analytical pipelines. Additionally, donor metadata was used for QC to ensure no sex- or donor-specific clusters in our cell type taxonomies.

Patch-seq:
Electrophysiological features were extracted without having information about the molecular typing of the cell. Cell type was determined by mapping patch-seq transcriptomic data onto corresponding species Cv3 or SSv4 reference dataset. Researchers were blinded to donor, but not species metadata during alignment.

Reporting for specific materials, systems and methods

We require information from authors about some types of materials, experimental systems and methods used in many studies. Here, indicate whether each material, system or method listed is relevant to your study. If you are not sure if a list item applies to your research, read the appropriate section before selecting a response.

Materials & experimental systems

n/a	Involved in the study	
☐	☒	Antibodies
☒	☐	Eukaryotic cell lines
☒	☐	Palaeontology and archaeology
☐	☒	Animals and other organisms
☐	☒	Human research participants
☒	☐	Clinical data
☒	☐	Dual use research of concern

Methods

n/a	Involved in the study	
☒	☐	ChIP-seq
☒	☐	Flow cytometry
☒	☐	MRI-based neuroimaging

Antibodies

Antibodies used

All data for this manuscript was collected in the set of corresponding core companion papers. All antibodies used in those studies are described in the core companion papers cited in the manuscript, also listed in the above "Data collection" section.

Validation

Animals and other organisms

Policy information about studies involving animals; ARRIVE guidelines recommended for reporting animal research

Laboratory animals

Epi-Retro-Seq: 42-49 day old adult male and female INTACT mice (R26R-CAG-loxp-stop-loxp-Sun1-sfGFP-Myc maintained on C57BL/6J background) were used for Epi-Retro-Seq experiments.

snmC-seq2: Adult (P56) C57BL/6J male mice

snATAC-seq: Adult (P56) C57BL/6J male mice

sc/snRNA-seq: Adult (P56) C57BL/6J male and female mice

Human, macaque, marmoset, mouse transcriptomics:
Common marmoset (Callithrix jacchus) animals were used (2 males, 1.9 years and 2.3 years; and 1 female, 3.1 years).
Pig-tailed macaque (Macaca nemestrina) animals were used (2 males, 12 and 17 years; and 1 female, 3 years).
Mouse (Mus musculus) animals were used (male and female wildtype C57Bl/6J P56 +/- 3 days).

MERFISH: Mus musculus, C57BL/6, male, P57-63

Mouse Patch-seq: Male and Female mice (median age 75 days, interquartile range 64-100, full range 35-245 days) were used in this study. Specific information about every single animal can be found in https://github.com/berenslab/mini-atlas. In particular, we used C57Bl/6 Wild type, Viaat-Cre/Ai9 mice, SOM-Cre/Ai9, VIPCre/Ai9, PV-Cre/Ai9, NPY-Cre/Ai9, Scl17a8-Cre/Ai9, Scl17a8-iCre/Ai9, Vipr2-Cre/Ai9 and Gnb4-Cre/Ai9. Detailed information about the origin of each single Cre line reported here can be find in the main text.

Anatomy: Mus musculus, male and female, 2-month old, wild type C57Bl6, Cre driver transgenics and reporters, some obtained from Jackson Laboratories.

Transgenic line generation and characterization: Mus musculus, mixed C57BL6 background (transgenes as indicated in text), 4-8 weeks age, mixed sex.

All rodent rooms are on a 12/12 hr light/dark cycle (6am - 6pm), except that Allen Institute rodent rooms are on a 14/10 hr light/dark cycle (6am-8pm). The room temperature range is 68-72°F (20-22°C) and the humidity range is 30-70%.

Wild animals

This study did not involve wild animals.

Field-collected samples

The study did not involve samples collected from the field.

Ethics oversight

All experimental procedures using live animals were performed according to protocols approved by Institutional Animal Care and Use Committees (IACUC) of all participating institutions: Allen Institute for Brain Science, Baylor College of Medicine, Broad Institute of MIT and Harvard, Cold Spring Harbor Laboratory, Harvard University, Salk Institute for Biological Studies, University of California Berkeley, University of California San Diego, University of Southern California. Macaque experiments were performed on animals designated for euthanasia via the Washington National Primate Research Center’s Tissue Distribution Program.

Human research participants

Policy information about studies involving human research participants

Population characteristics

Human transcriptomics:
43 y/o Iranian female with PMI 18.5 hours from mitral valve prolapse (SSv4),
50 y/o caucasian male with PMI 24.5 hours from cardiovascular event (SSv4),
54 y/o caucasian male with PMI 25 hours from cardiovascular event (SSv4),
60 y/o unknown female with PMI 18 hours from car accident (SSv4, Cv3, SNARE-seq2, snmC-seq2),
and 50 y/o unknown male with PMI 10 hours from cardiovascular event (SSv4, Cv3, SNARE-seq2, snmC-seq2).

Data type: SMART-Seqv4 (SSv4), 10x Genomics Chromium Single Cell 3’ Kit v3 (Cv3), Single-Nucleus Chromatin Accessibility and mRNA Expression sequencing (SNARE-seq2), Single nucleus methyl cytosine sequencing (snmCseq2).

Human Patch-seq:
61 y/o caucasian female undergoing surgical resection for treatment of deep brain tumor.

Recruitment

Human transcriptomics:
Postmortem adult human brain tissue was collected after obtaining permission from decedent next-of-kin. Postmortem tissue specimens from males and females between 18 – 68 years of age with no known history of neuropsychiatric or neurological conditions (‘control’ cases) were considered for inclusion in this study of cell transcriptional profiles. Key conditions for exclusion were:

- Known brain injury, cancer or disease
- Known neuropsychiatric or neuropathological history
- Epilepsy or other seizure history
- Drug/alcohol dependency
- > 1 hour on ventilator
- Positive for infectious disease
- Prion disease
- Chronic renal failure
- Death from homicide or suicide
- Sleep apnea
- Time since death (postmortem interval, PMI) > 25 hours

Human Patch-seq:
Tissue was collected after obtaining informed consent of the patient

Ethics oversight

Human transcriptomics:
Postmortem adult human brain tissue collection was performed in accordance with the provisions of the United States Uniform Anatomical Gift Act of 2006 described in the California Health and Safety Code section 7150 (effective 1/1/2008) and other applicable state and federal laws and regulations. The Western Institutional Review Board reviewed tissue collection processes and determined that they did not constitute human subjects research requiring institutional review board (IRB) review.

Human Patch-seq:
The patient provided informed consent and experimental procedures were approved by the hospital institute review board before commencing the study.

Note that full information on the approval of the study protocol must also be provided in the manuscript.
